# Mechanisms of the Photomechanical Response in Thin-Film Dye-Doped Glassy Polymers

**DOI:** 10.3390/polym17020254

**Published:** 2025-01-20

**Authors:** Zoya Ghorbanishiadeh, Ankita Bhuyan, Bojun Zhou, Morteza Sheibani Karkhaneh, Mark G. Kuzyk

**Affiliations:** Department of Physics, Washington State University, Pullman, WA 99163, USA; z.ghorbanishiadeh@wsu.edu (Z.G.); ankitaccg@gmail.com (A.B.); bojun.zhou@hotmail.com (B.Z.); morteza.sheibani.2016@gmail.com (M.S.K.)

**Keywords:** dye-doped polymers, rheology, thermal expansion, photomechanics, photothermal heating, orientational hole burning, molecular reorientation, dichroism, thin films, Young’s modulus, photomechanical efficiency

## Abstract

This work aims to determine the mechanism of the photomechanical response of poly(Methyl methacrylate) polymer doped with the photo-isomerizable dye Disperse Red 1 using the non-isomerizable dye Disperse Orange 11 as a control to isolate photoisomerization. Samples are free-standing thin films with thickness that is small compared with the optical skin depth to assure uniform illumination and photomechanical response throughout their volume, which differentiates these studies from most others. Polarization-dependent measurements of the photomechanical stress response are used to deconvolute the contributions of angular hole burning, molecular reorientation and photothermal heating. While photo-isomerization of dopant molecules is commonly observed in dye-doped polymers, the shape changes of a molecule might not couple strongly to the host polymer through steric mechanical interactions, thus not contributing substantially to a macroscopic shape change. To gain insights into the effectiveness of such mechanical coupling, we directly probe the dopant molecules using dichroism measurements simultaneously while measuring the photomechanical response and find mechanical coupling to be small enough to make photothermal heating—mediated by the transfer of optical energy as heat to the polymer—the dominant mechanism. We also predict the fraction of light energy converted to mechanical energy using a model whose parameters are thermodynamic material properties that are measured with independent experiments. We find that in the thin-film geometry, these dye-doped glassy polymers are as efficient as any other material but their large Young’s modulus relative to other organic materials, such as liquid crystal elastomers, makes them suitable in applications that require mechanically strong materials. The mechanical properties and the photomechanical response of thin films are observed to be significantly different than in fibers, suggesting that the geometry of the material and surface effects might play an important role.

## 1. Introduction

**Figure 1:**
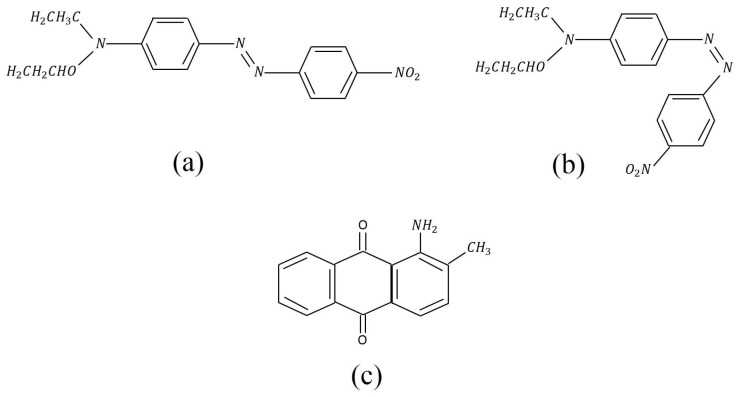
Molecular structure of (**a**) the trans state of Disperse Red 1 (DR1), (**b**) the cis form, and (**c**) Disperse Orange 11 (DO11) [2].

Photoisomerization is a reversible process where a molecule’s conformation changes upon absorbing a photon. Underlying processes in azobenzene chromophores include trans–cis conversion and an open–closed ring transition [1]. Such molecules, when doped into a polymer, will induce a bulk photomechanical response. Figure 1 shows the Disperse Red 1 (DR1) molecule, which is a commercially available azobenzene dye that we use in our studies.

The trans form, being of lower energy, is dominant at low temperatures and in the dark. Certain wavelengths of light can excite the trans molecule into the cis state, which can subsequently relax nonradiatively to the trans state or the de-excitation can be induced by absorbing a photon [1,3]. This specific switchable property of azo dyes has attracted considerable attention for their promise in novel applications such as optical actuation, refs. [4,5,6,7,8]; optical data storage, refs. [9,10,11]; all-optical switching, ref. [12]; holography, ref. [13]; grating fabrication, refs. [14,15]; and nonlinear optical effects [16,17,18].

Photomechanical materials convert light energy into mechanical work [1,3,4]. Two dominant mechanisms include photothermal heating, where the light energy absorbed by the dopant molecules is transferred as heat to the polymer, hence increasing the material’s temperature and inducing thermal expansion, and photoisomerization, where through steric interaction dye dopants stress the surrounding polymer when changing shape or reorienting [1,4,19]. Common methods to study photoisomerization and photo-(re)orientation include photo-induced birefringence and dichroism [10,20,21,22,23]. However, changes in molecular shape and orientation might not result in a photomechanical effect of the bulk polymer. An understanding of the mechanical coupling efficiency of the molecule to the host polymer thus requires experiments that both probe the dopant molecules and measure the bulk photomechanical response. Past studies using second harmonic generation to probe molecular reorientation of a dye molecule in a polymer matrix suggested weak mechanical coupling based on the high-level of molecular mobility [24]. However, there have been no direct measurements of the effect on the photomechanical response.

Kawabe and Okoshi separated the contribution of angular hole burning and molecular reorientation using dichroism and birefringence in DR1-doped PMMA thin films as a function of dye concentration. They measured the absorption spectra before, during, and after illuminating the sample with a polarized pump in the presence of a probe beam. They also probed the sample’s birefringence and found that the light absorbance along and perpendicular to the pump polarization decreases with the perpendicular polarization dropping more than the parallel one. Kawabe and Okoshi concluded that angular hole burning is a fast process that dominates dichroism at short exposure times, and molecular reorientation becomes notable only after a longer exposure time and is stronger in samples with higher dye concentration [23]. Rodriguez et al., on the other hand, found that the birefringence and absorption coefficient decreased significantly parallel to the pump polarization, while the reduction was negligible in the perpendicular direction, ref. [22], indicative of hole burning. In contrast to these studies, our work uses thin freestanding films whose optical skin depth is much larger than the thickness, yielding uniform illumination for a more accurate determination of the population’s orientational dynamics and a more uniform photomechanical force. In addition, our experiments also simultaneously measure the photomechanical response so that molecular reorientation can be correlated with changes in the orientation distribution function and relative isomer populations.

Silva et al. studied V-shaped azo-chromophores doped into PMMA thin films as a function of dye concentration to investigate their optical storage performance. They observed a decrease in the absorption spectra when the probe light is polarized parallel to the pump and an increase when the polarization is perpendicular to the pump. They conclude that molecular reorientation dominates angular hole burning in their materials. The long-lasting residual persistence of the angular hole due to reorientation was investigated for its potential in optical memory. The high quantum efficiency of photo-induced birefringence is related to angular hole burning due to a change in isomer populations [10]. These processes at the molecular level can lead to a bulk photomechanical response if the molecules couple to the polymer host, so understanding the molecular process is an important step in optimizing materials for optical actuation applications [1,4,25].

The present work combines (1) new theory development, (2) a unique experimental design that simultaneously measures the photomechanical tensor response while probing with optical dichroism as a function of time during optical pumping and (3) a free-standing thin-film sample that can be uniformly illuminated without the constraints of a substrate. Each of these alone differentiates our approach from most others. The goal of the work is to determine the relative contributions of photo-heating, photoisomerization, molecular reorientation and angular hole burning to the photomechanical stress response. Measurements include light-induced dichroism and stress as a function of time, intensity and temperature in DR1-doped PMMA (DR1/PMMA) thin films using Disperse Orange 11-doped PMMA (DO11/PMMA) thin films as a control. DO11 is the ideal control because it does not photoisomerize and its optical absorption spectrum is similar to DR1, making it possible to match the optical absorbance of the two dyes at the measurement wavelength by controlling the relative concentrations. The wealth of data obtained, along with a measurement of Young’s modulus, allows us to determine the photomechanical figure of merit, which quantifies the efficiency of converting a photon’s energy to mechanical work. The understanding gained from studies of the various mechanisms will inform the development of more efficient materials for applications that require the direct conversion of light to work.

## 2. Theory

This section develops the models that will be used in the analysis of the data. There are many separate and independent theoretical parts to this work. We start with an overview here to prepare the reader with a broader overview.

The start of Section 2.1 defines the constitutive relations of the photomechanical response. The important result is that the photomechanical stress is a function of the pump intensity that illuminates the material, which is well approximated by a power series in the field. Secondly, the stress response varies in time after the pump light is turned on, which we assume takes on an exponential form because it fits the data well.

Section 2.1.1 focuses solely on a model for photothermal heating, where light energy absorbed by the dye molecules is transferred to the host polymer as heat. The central idea is that the heating response is independent of the heat source, be it light or hot air in an oven. First, a phenomenological model for the stress as a function of temperature is introduced that fits the data for a film whose length is constrained. Then, the section ends with determining the photomechanical coefficients due to heat by relating the temperature increase to the pump light’s intensity.

Section 5.1.2 relates the stress induced by two orthogonal pump polarizations relative to the principal sample axis to the two tensor components of the photomechanical stress tensor. The various mechanisms have distinct photomechanical stress tensor ratios, so a measure of the stress induced by two orthogonal pump polarizations is a metric of the mechanisms.

The theoretical results will be used to fit and interpret the data. The reader who is interested in the experimental results can skip the theory section.

### 2.1. Photomechanical Response

The stress response of a material to light is a function of the intensity. For small enough intensities, most functions can be approximated by a power series expansion of the form

(1) $$\begin{array}{c} \sigma \left(t\to \infty \right)=-\sum_{n=1}^{\infty }\kappa_{\sigma }^{\left(n\right)}I^{n}\approx -\left(\kappa_{\sigma }^{\left(1\right)}I+\kappa_{\sigma }^{\left(2\right)}I^{2}\right), \end{array}$$

where σ is the stress, $\kappa_{\sigma }^{\left(n\right)}$ the $n^{\mathrm{th}}$-order photomechanical coefficient and *I* the absorbed intensity [1,4]. The first two terms in the expansion are found to model typical materials in the intensity ranges measured. If the material is mechanically stressed, there would also be a term corresponding to n=0, but this would not be a material property but rather an experimental constraint. Equation (1) thus describes a material in its relaxed state. The photomechanical coefficients will be different in an initially stressed material, but we will assume that all pre-stress due to sample mounting will be small enough to ignore this effect.

When $\kappa_{\sigma }^{\left(n\right)}$> 0, light causes the sample’s equilibrium length to increase. When the sample length is held constant by the clamps, the material becomes compressed if it is not to buckle. By definition, a compressional force that opposes expansion is negative, so σ<0. The experiment measures this stress—defined to be along the *z*-direction—as a function of time after the pump light (measured separately for each pump polarization) is turned on.

The photomechanical stress response as a function of time for fixed pump intensity is assumed to follow a bi-exponential function of the form

(2) $$\begin{array}{cc} \sigma_{\mathrm{on}}\left(t\right) & =\sigma_{0}+\sigma_{1}\left(1-e^{\left(-t/t_{1}\right)}\right)+\sigma_{2}\left(1-e^{\left(-t/t_{2}\right)}\right), \end{array}$$

and

(3) $$\begin{array}{cc} \sigma_{\mathrm{off}}\left(t\right) & =\sigma_{0}+\sigma_{1}\left(1-e^{\left(-t_{0}/t_{1}\right)}\right)e^{-\left(t-t_{0}\right)/t_{3}}\\ \; & +\sigma_{2}\left(1-e^{\left(-t_{0}/t_{2}\right)}\right)e^{-\left(t-t_{0}\right)/t_{4}}. \end{array}$$

**Figure 2:**
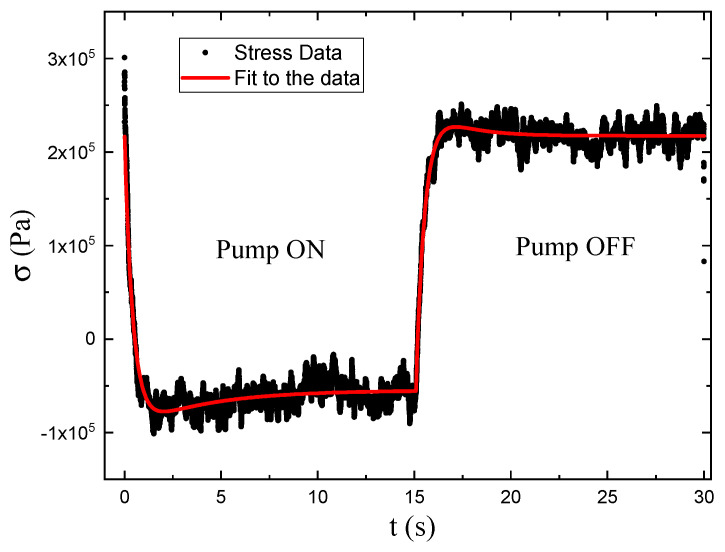
Typical photomechanical stress response as observed in a film as a function of time and a fit to the theory given by Equations (2) and (3).

The parameters $t_{n}$ are time constants, $\sigma_{n}$ the amplitudes and $t_{0}$ is the time offset between pump turn-on and turn-off [4]. Figure 2 shows typical data and fits to a bi-exponential as given by Equations (2) and (3).

#### 2.1.1. Heating

The photothermal heating mechanism is a two-step process that converts the absorbed light energy into heat, which increases the material’s temperature and induces thermal expansion. Direct heating in an oven should yield the same amount of expansion for the same amount of deposited energy; the source of heat is irrelevant.

We assume that the temperature dependence of stress is of the form

(4) $$\begin{array}{c} \sigma \left(T\right)=\sigma_{A}+\frac{\sigma_{B}}{\left(1+{\left(\frac{T}{T_{0}}\right)}^{n}\right)}. \end{array}$$

**Figure 3:**
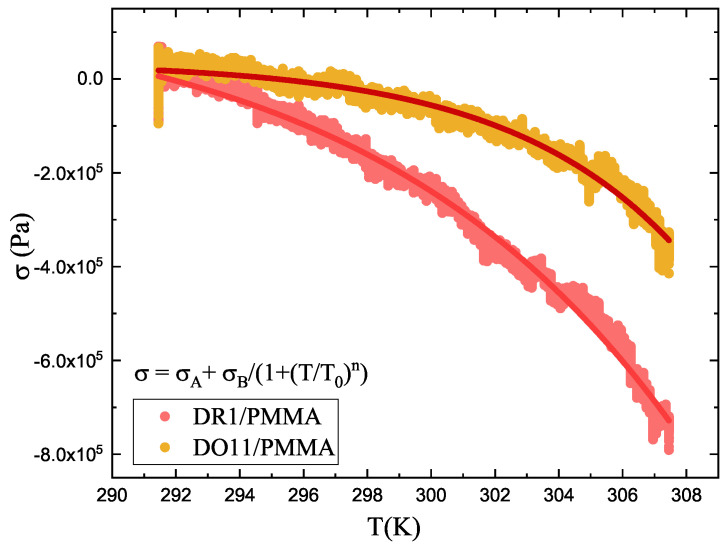
Measured temperature-dependent stress at constant sample length and with no light applied of DR1 and DO11 thin films (points) and a fit of the data (curves) to Equation (4).

Here, $\sigma_{A}$ is the pre-stress due to sample mounting and is not related to the material property, *n* an exponent, $T_{0}$ a critical temperature and $\sigma_{B}$ the strength of the temperature-dependent response. Note that these parameters may have physical meaning, but this function and set of parameters are just a guess and are based on the fact that they fit the data well and will thus allow for an analytical analysis of the data using one simple equation. Figure 3 shows a fit to the data as an example, which will be described in greater detail later. For example, we can determine the change in stress per change in temperature simply by taking the derivative of this analytical form and using it to determine the thermal photomechanical stress response coefficients at any temperature. Any functional form that fits the data can be used as the starting point.

The connection of the temperature-dependent stress to the thermal photomechanical response originates in the temperate change ΔT induced by the heat deposited by the light of intensity *I*, given by [2]

(5) $$\begin{array}{c} \Delta T=\frac{\tau }{c\rho t}I, \end{array}$$

where τ is the time it takes to reach steady state after the pump light is turned on, where the energy absorbed from the light balances the heat radiated from the sample, ρ is the density, *c* the specific heat and *t* the sample thickness.

The approach is to evaluate Equation (4) at T=T+ΔT where ΔT is given by Equation (5) and then expand the function for small intensity such that ΔT≪T. The stress as a function of intensity is then given by the series expansion

(6) $$\begin{array}{cc} \sigma (T,I) & =\sigma \left(T,\frac{\tau }{c\rho t}I\right)=\sigma \left(T,0\right)+{\left.\frac{\partial \sigma }{\partial T}\right|}_{T,I=0}\frac{\tau }{c\rho t}I\\ \; & +\frac{1}{2}{\left.\frac{\partial^{2}\sigma }{\partial T^{2}}\right|}_{T,I=0}{\left(\frac{\tau }{c\rho t}\right)}^{2}I^{2}+\ldots , \end{array}$$

where we have expanded the stress as a series of *I* under the assumption that the temperature change due to the stress is small compared with the ambient temperature.

The change in stress with temperature, given by the derivative of Equation (4), yields

(7) $$\begin{array}{c} \frac{d\sigma }{dT}=-\frac{\frac{n\sigma_{B}}{T}{\left(\frac{T}{T_{0}}\right)}^{n}}{{\left(1+{\left(\frac{T}{T_{0}}\right)}^{n}\right)}^{2}} \end{array}$$

and the second derivative is given by

(8) $$\begin{array}{c} \frac{d^{2}\sigma }{dT^{2}}=-\frac{n\sigma_{B}}{T^{2}}{\left(\frac{T}{T_{0}}\right)}^{2n}\cdot \frac{\left(n-1\right)-\left(n+1\right){\left(\frac{T}{T_{0}}\right)}^{n}}{{\left(1+{\left(\frac{T}{T_{0}}\right)}^{n}\right)}^{3}}. \end{array}$$

Substituting Equations (7) and (8) into Equation (6), we can read off the photomechanical stress response coefficients, yielding

(9) $$\begin{array}{c} \kappa_{\sigma }^{\left(1\right)}\left(T\right)=\sigma_{B}\frac{\frac{n\tau }{c\rho tT}{\left(\frac{T}{T_{0}}\right)}^{n}}{{\left(1+{\left(\frac{T}{T_{0}}\right)}^{n}\right)}^{2}} \end{array}$$

and

(10) $$\begin{array}{c} \kappa_{\sigma }^{\left(2\right)}\left(T\right)=2n\sigma_{B}{\left(\frac{\tau }{c\rho t}\right)}^{2}\frac{1}{T^{2}}{\left(\frac{T}{T_{0}}\right)}^{2n}\cdot \frac{\left(n-1\right)-\left(n+1\right){\left(\frac{T}{T_{0}}\right)}^{n}}{{\left(1+{\left(\frac{T}{T_{0}}\right)}^{n}\right)}^{3}}. \end{array}$$

When the photomechanical response is well approximated as a quadratic function of the intensity, we can define an effective intensity-dependent photomechanical stress response coefficient $\kappa_{\sigma }^{\left(1\right)}\left(I\right)$ given by

$$\kappa_{\sigma }^{\left(1\right)}\left(I,T\right)=\kappa_{\sigma }^{\left(1\right)}\left(T\right)+\kappa_{\sigma }^{\left(2\right)}\left(T\right)I$$

(11) $$=\sigma_{B}\frac{\frac{n\tau }{c\rho tT}{\left(\frac{T}{T_{0}}\right)}^{n}}{{\left(1+{\left(\frac{T}{T_{0}}\right)}^{n}\right)}^{2}}+2n\sigma_{B}{\left(\frac{\tau }{c\rho t}\right)}^{2}\frac{1}{T^{2}}{\left(\frac{T}{T_{0}}\right)}^{2n}\cdot \frac{\left(n-1\right)-\left(n+1\right){\left(\frac{T}{T_{0}}\right)}^{n}}{{\left(1+{\left(\frac{T}{T_{0}}\right)}^{n}\right)}^{3}}I.$$

Here, we pause to stress a point of potential confusion regarding the signs. Recall that the negative sign in Equation (1) is introduced because of the convention that the sample length increases when the photomechanical constant is positive, which leads to a decrease in the stress when the sample is in the clamped configuration. In the clamped experiment, where the material is pre-stressed, the magnitude of the measured force will decrease when the sample expands. Hence, a negative slope of the stress versus temperature is an indicator of thermal expansion. To summarize, the negative sign in Equation (1) requires that the sign of Equation (9) be opposite to the sign of Equation (7).

Alternatively, we can eliminate the parameter $\sigma_{B}$ in Equation (9) using Equation (4), yielding the semi-empirical model [2]

(12) $$\begin{array}{c} \kappa_{\sigma }^{\left(1\right)}\left(I\right)=\frac{\tau }{\rho ct}\cdot \frac{n(\sigma -\sigma_{A})}{T\left(1+{\left(\frac{T_{0}}{T}\right)}^{n}\right)}. \end{array}$$

This model is semi-empirical in that σ(I), the stress in the limit of infinite time after the pump of intensity *I* is turned on, is an experimentally determined value. So, we emphasize that in the semi-empirical approach, we are using the measured stress induced by the light rather than by a temperature change because of the one-to-one correspondence between temperature change and the intensity, which is the source of the deposited heat. The pre-stress, called $\sigma_{0}$ in the photomechanical measurements and $\sigma_{A}$ in the temperature-dependent stress, is the initial stress from the sample being mounted under tension. **Thus, the empirically determined intensity-dependent time constant τ(I) and intensity-dependent σ(I) together are responsible for the intensity dependence of $\kappa_{\sigma }^{\left(1\right)}\left(I\right)$.**

In principle, the time constant and stress for the heating mechanism should be independent of the intensity. However, a substantial increase in the sample’s temperature can change its elastic properties and thus lead to a nonlinearity that yields an intensity-dependent photomechanical constant. **Therefore, a discrepancy between the zero-intensity photomechanical constant and the heating theory is an indicator of other mechanisms at play.** Below, we start with comparisons of the temperature derivatives of the stress as a proxy for photothermal heating. Later in the paper, we will fit the time-dependent stress to a bi-exponential and apply the heating theory to the time constants and stresses so determined to isolate the process that corresponds to photothermal heating.

Since the samples are prepared to be isotropic, and below we will see this to be approximately true, the heating rate should be independent of the polarization of the light and thermal expansion should also be isotropic. If the photomechanical response is due solely to heating, we expect the linear photomechanical stress response to be given by Equation (9) or (12), which assumes that the absorbed heat leads to a temperature change that drives thermal expansion [4].

The heating theory presented here is a zeroth-order approximation of a more comprehensive theory. For example, a material that undergoes photo-isomerization will result in a change in the amount of light absorbed due to hole burning even if hole burning does not directly lead to a photomechanical effect. Hole burning will, however, change the amount of heat transferred to the material, which is not accounted for in this theory. Other issues include the following: (1) This is a low-temperature theory that only applies below the phase transition temperature. (2) The presence of dopant dyes with two or more conformations affect the heat capacity and thermal expansion coefficient by adding additional degrees of freedom that need to be taken into account. This will be evident by the large difference in the thermally induced stress between DO11 and DR1 dye doping as described later. The present theory will be sufficient to interpret our data, and the more refined theory of the contribution of photothermal heating is under development and will be the topic of a future paper.

#### 2.1.2. Hole Burning and Photo-Stress Anisotropy

Light-induced angular hole burning, which is commonly observed in azo-dye-doped polymers such as PMMA/DR1, induces an anisotropic photomechanical stress response [1,2]. Hole burning generally refers to the process where an originally isotropic material, represented by a spherical orientational distribution, develops an anisotropy like the dimples on an apple’s north and south poles. These dimples represent a depletion of molecules orientated in that direction and therefore called an orientational hole. There are many processes that can lead to such a hole, such as the molecules aligned along the light’s polarization axis being excited and molecules diffusing away from the light’s polarization axis. The former is usually referred to as angular hole burning, while the latter is called molecular reorientation.

Assuming that the photomechanical response is a linear function of the intensity, as observed in Figure 6, the ratio of stress for two orthogonal pump polarizations is given by [1,2]

(13) $$\begin{array}{c} \frac{\sigma_{//}}{\sigma_{⊥}}=\frac{-\kappa_{//}^{\left(1\right)}I}{-\kappa_{⊥}^{\left(1\right)}I}=\frac{\kappa_{//}^{\left(1\right)}}{\kappa_{⊥}^{\left(1\right)}}=\frac{\kappa_{//}^{\left(\mathrm{heat}\right)}+\kappa_{//}^{\left(\mathrm{hole}\right)}}{\kappa_{⊥}^{\left(\mathrm{heat}\right)}+\kappa_{⊥}^{\left(\mathrm{hole}\right)}}, \end{array}$$

where the stress response from the two mechanisms is assumed to be additive. Note that the parallel stress $\sigma_{//}$ is defined to be the stress induced for a pump beam that is polarized along the direction of the stress measurement while $\sigma_{⊥}$ is perpendicular to it. Note that the superscript “hole” refers to both angular hole burning and molecular reorientation. Once the heating contribution is isolated, these two mechanisms can be separated.

The photothermal heating contribution for an isotropic material is independent of polarization, so

(14) $$\begin{array}{c} \kappa_{//}^{\left(\mathrm{heat}\right)}=\kappa_{⊥}^{\left(\mathrm{heat}\right)}\equiv \kappa^{\left(\mathrm{heat}\right)}\left(I\right). \end{array}$$

Polarized light acting on randomly oriented azo dyes doped into a polymer induces reorientational diffusion away from the light’s polarization. This depletion of molecules oriented along the light’s polarization results in a decreased length along the light’s polarization and an increase perpendicular to it. If the orientational distribution function retains azimuthal symmetry, conservation of volume demands that

(15) $$\begin{array}{c} \kappa_{//}^{\left(\mathrm{hole}\right)}=-2\kappa_{⊥}^{\left(\mathrm{hole}\right)}\equiv \kappa^{\left(\mathrm{hole}\right)}\left(I\right). \end{array}$$

Using Equations (14) and (15), Equation (13) becomes

(16) $$\begin{array}{c} \frac{\sigma_{//}}{\sigma_{⊥}}=\frac{\kappa_{//}^{\left(1\right)}}{\kappa_{⊥}^{\left(1\right)}}=\frac{\kappa^{\left(\mathrm{heat}\right)}\left(I\right)+\kappa^{\left(\mathrm{hole}\right)}\left(I\right)}{\kappa^{\left(\mathrm{heat}\right)}\left(I\right)-\frac{1}{2}\kappa^{\left(\mathrm{hole}\right)}\left(I\right)}=\frac{1+\frac{\kappa^{\left(\mathrm{hole}\right)}\left(I\right)}{\kappa^{\left(\mathrm{heat}\right)}\left(I\right)}}{1-\frac{1}{2}\frac{\kappa^{\left(\mathrm{hole}\right)}\left(I\right)}{\kappa^{\left(\mathrm{heat}\right)}\left(I\right)}}. \end{array}$$

Since we seek to determine the relative contribution of the two mechanisms, we define their ratio as *R* and invert Equation (16) to obtain

(17) $$\begin{array}{c} R\equiv \frac{\kappa^{\left(\mathrm{hole}\right)}\left(I\right)}{\kappa^{\left(\mathrm{heat}\right)}\left(I\right)}=\frac{\frac{\kappa_{//}^{\left(1\right)}\left(I\right)}{\kappa_{⊥}^{\left(1\right)}\left(I\right)}-1}{\frac{1}{2}\frac{\kappa_{//}^{\left(1\right)}\left(I\right)}{\kappa_{⊥}^{\left(1\right)}\left(I\right)}+1}. \end{array}$$

If the only two acting mechanisms are photothermal heating and angular hole burning, which includes both changes in the isomer populations and molecular reorientation, all of the photomechanical properties of the material can be expressed in terms of two parameters. As such, two measurements (e.g., the photo-induced stress measured for two orthogonal pump polarizations) are sufficient to determine all others. For example, Equations (15) and (17) yield

(18) $$\begin{array}{c} \kappa_{//}^{\left(\mathrm{hole}\right)}=R\kappa^{\left(\mathrm{heat}\right)}\left(I\right), \end{array}$$

Therefore, for stress induced parallel to the light’s polarization, the linear photomechanical constant is given by

(19) $$\begin{array}{c} \kappa_{//}^{\left(1\right)}=\kappa_{//}^{\left(\mathrm{heat}\right)}+\kappa_{//}^{\left(\mathrm{hole}\right)}=\left(1+R\right)\kappa^{\left(\mathrm{heat}\right)}\left(I\right) \end{array}$$

So, in this example, *R* and $\kappa^{\left(\mathrm{heat}\right)}\left(I\right)$ are the independent parameters.

Conversely, Equations (18) and (19) can be inverted to determine the relative strength of the mechanisms, yielding

(20) $$\begin{array}{c} \kappa^{\left(\mathrm{heat}\right)}\left(I\right)=\frac{\kappa_{//}^{\left(1\right)}}{1+R}, \end{array}$$

and

(21) $$\begin{array}{c} \kappa^{\left(\mathrm{hole}\right)}\left(I\right)=\frac{R\kappa_{//}^{\left(1\right)}}{1+R}. \end{array}$$

Similarly, it is trivial to show that

(22) $$\begin{array}{c} \kappa_{//}^{\left(\mathrm{heat}\right)}=\kappa_{⊥}^{\left(\mathrm{heat}\right)}=\kappa^{\left(\mathrm{heat}\right)}\left(I\right)=\frac{\kappa_{//}^{\left(1\right)}}{1+R}, \end{array}$$

(23) $$\begin{array}{c} \kappa_{//}^{\left(\mathrm{hole}\right)}=\frac{R\kappa_{//}^{\left(1\right)}}{1+R}, \end{array}$$

and

(24) $$\begin{array}{c} \kappa_{⊥}^{\left(\mathrm{hole}\right)}=-\frac{1}{2}\frac{R\kappa_{//}^{\left(1\right)}}{1+R}. \end{array}$$

For the second order, Equation (17) can be generalized to obtain the ratio *R* as a function of intensity from

(25) $$\begin{array}{c} \frac{\sigma_{//}\left(I\right)}{\sigma_{⊥}\left(I\right)}=\frac{\kappa_{//}\left(I\right)}{\kappa_{⊥}\left(I\right)}=\frac{\kappa_{//}^{\left(1\right)}I+\kappa_{//}^{\left(2\right)}I^{2}}{\kappa_{⊥}^{\left(1\right)}I+\kappa_{⊥}^{\left(2\right)}I^{2}}, \end{array}$$

which yields

(26) $$\begin{array}{c} R=\frac{\kappa^{\left(\mathrm{hole}\right)}\left(I\right)}{\kappa^{\left(\mathrm{heat}\right)}\left(I\right)}=\frac{\frac{\kappa_{//}^{\left(1\right)}+\kappa_{//}^{\left(2\right)}I}{\kappa_{⊥}^{\left(1\right)}+\kappa_{⊥}^{\left(2\right)}I}-1}{\frac{1}{2}\frac{\kappa_{//}^{\left(1\right)}+\kappa_{//}^{\left(2\right)}I}{\kappa_{⊥}^{\left(1\right)}+\kappa_{⊥}^{\left(2\right)}I}+1}. \end{array}$$

## 3. Approach

The Disperse Red 1 (DR1) and Disperse Orange 11 (DO11) dyes used in our studies are shown in Figure 1. The azo bond (–N=N–) changes conformation upon heating [1,3] or absorbing light [1], while DO11 remains mostly unchanged. The sole mechanism in DO11 should be a photothermal response (aside from photo tautomerization [26,27]—which is not associated with a detectable photomechanical effect [1]). As such, DO11 is used as a control to determine the relative contributions of photoisomerization and photothermal heating in DR1 by comparing samples of the two materials with dye concentrations that absorb the same amount of light.

The smallest photomechanical part of a material whose properties are the same as the material is called a photomorphon [1,4]. The photomorphon, in turn, is made of the photomechanical unit (usually the active dye molecule) along with the passive material surrounding it. In a dye-doped polymer, a single dopant molecule is the photomechanical unit and with the surrounding polymer makes the photomorphon. The photomechanical unit can be placed in series and/or in parallel with the photomechanical unit, depending on the structure of the polymer.

In an isotropic material, the amount of heat deposited is independent of the light’s polarization. Thus, the measured light-induced stress from thermal expansion along the direction the sample is clamped is independent of the polarization of the light. Angular hole burning refers to the process by which molecules that are aligned along the light’s polarization are converted to a more compact and more-nearly spherical isomer, thus inducing anisotropy in the material. This anisotropy leads to both an anisotropic refractive index/absorbance and stress. “Angular hole burning” is a relatively fast process compared with the other mechanisms. The slower molecular reorientational process, which also leads to anisotropy, results from a depletion of molecules oriented along the light’s polarization axis and an increase perpendicular to it, yielding anisotropy in the stress and refractive index/absorbance [1,2,4], which builds over longer time scales. While this is also technically “angular hole” burning due to the depletion of molecules oriented along the pump polarization, it is called “molecular reorientation” to differentiate it from the faster process and to stress that the molecules are reorienting away from the light’s polarization. As described later, the characteristics of these two types of anisotropies can be used to deconvolute the relative contributions of photo-thermal heating, angular hole burning and reorientation.

In materials with azo-dye molecules, molecular reorientation away from the light’s polarization is mediated by the preferential absorption of light by trans molecules that are aligned with the light’s polarization, followed by rotational diffusion of the more compact and more mobile cis form. The higher-energy cis isomers that are formed, upon de-excitation to the trans form, result in a random distribution of orientation, leaving a depleted trans population along the light’s polarization axis [1,2,4,28,29,30]. Generally, molecular reorientation results in both the length of the sample and the absorbance along the light’s polarization axis to decrease. They both increase in the plane perpendicular to the polarization axis.

In addition to studying the mechanisms of the photomechanical response, we also evaluate the figure of merit (FOM), which quantifies the efficiency of a material in converting light energy to mechanical work. This gives an estimate of the efficiency of photoisomerization (DR1) and photothermal heating (DO11). The thin films used in our experiments are specifically tailored to absorb light uniformly so that the photomechanical stress response $\kappa_{\sigma }$ can be determined accurately. Along with Young’s modulus *E*, the material figure of merit is given by [1,2,4]

(27) $$\begin{array}{c} FOM=\frac{{\left(\kappa_{\sigma }^{\left(1\right)}\right)}^{2}}{E}. \end{array}$$

We note that Equation (27) assumes that Young’s modulus is unaffected by light to simplify the required set of measurements to obtain the FOM.

## 4. Experiment

### 4.1. Sample Preparation

Dye-doped polymeric thin-film samples are formed by spin coating, starting with solutions of Propylene Glycol Methyl Ether Acetate (PGMEA) and γ-Butyrolactone co-solvents and PMMA with either DR1 or DO11 dye solutes. The samples’ optical absorbances at λ = 488 nm are matched using 2.76 times more DO11 than DR1 dye in PMMA polymer by weight [4]. We use 0.5 weight % DR1 and 1.38 weight % DO11 as a percentage of PMMA. The DR1 solutions are composed of 7.33 mL of PGMEA, 0.5 W% DR1, 3.1 mL γ-Buterolactone and 1.8565 g PMMA. For DO11 solutions, we use the same solvents with 1.38 W% DO11 and 1.8491 g PMMA. The solutions are stirred overnight to fully dissolve the solids. Subsequently, the solution is transferred to a new clean bottle using a filtering syringe to remove any remaining residues. The filtered solution is refrigerated overnight before spin coating.

A vacuum chuck securely holds a clean glass substrate horizontal. The spin speed and spin time are set and the substrate flooded with a solution with a sterile syringe. The samples are spun for a fixed time and speed (typically 1000 to 10,000 RPM for 30 s), forming films in the thickness range of 1 to 10 micron. The coated substrates are placed in an oven at 90 °C for 30 min to insure that all of the solvent is removed. After being removed from the oven, the sample is submerged in distilled water to delaminate the film from the substrate.

### 4.2. Setup

**Figure 4:**
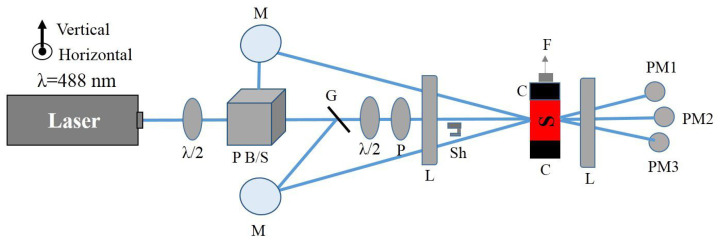
The apparatus used for the intensity and polarization-dependent response measurement. M: Mirror, P B/S: Polarized beam-splitter, G: Glass slide, P: Polarizer, λ/2: Half-wave plate, L: Lens, Sh: Shutter, C: Clamp, S: Sample, F: Force sensor, PM1, PM2, PM3: Power meters.

Figure 4 shows a schematic diagram of our experiment, which simultaneously measures the light-induced stress and monitors the transmittance of two orthogonal probe beams to determine the evolution of the population upon irradiation. The λ = 488 nm wavelength light from an argon laser passes through a half-wave plate and a polarizing beam splitter (PBS), then illuminates the sample. This beam acts as the pump, which induces a photomechanical response. The polarizer (*P*) is set to select the polarization of the pump light and the preceding half-wave plate is rotated to adjust its intensity. In a typical experiment, a run consists of the polarizer set to one angle, followed by a run where it is set perpendicular to the original orientation to obtain the polarization dependence of the response due to the pump along the two principal axes of the material.

Two orthogonally polarized probe beams are derived from the same laser. One of them is diverted from the rejected polarization by the PBS, while the other one is picked off by the glass slide *G* downstream from the PBS. Both probe beams and the pump beam pass through the same lens and fully overlap at the lens’ focus at the same point in the sample. The two probe beams’ polarizations are orthogonal to each other and set along the principal axes of the material, which are vertical and horizontal in our experiment. The pump polarization aligns with one or the other probe beam’s polarization.

The pump light induces a physical change in the sample, which results in a stress that is detected with a force sensor placed in series with the sample and attached to the upper clamp. The probe beam powers are much lower than the pump power, so the probe beams induce a negligible force. They remain on at all times and are monitored with photo detectors, which are interfaced with an Arduino and custom circuitry [31]. The pump beam is unblocked/blocked every 15 s with an automatic shutter (Sh). The transmitted pump light is also monitored with a photodetector. All intensities and the force are simultaneously recorded.

The pump light is first polarized vertically for a sequence of runs over time for several intensities so that the intensity-dependent photomechanical response and light-induced dichroism can be simultaneously determined. The same experiments are then run for the horizontal pump. This set of data determines the evolution of angular hole burning as determined from the probe beams and correlated with the photomechanical response. DR1-doped PMMA thin films are compared with a DO11 thin film as a control due to it not having multiple conformations. All samples are pre-stressed in the force-sensing clamp before starting the experiments to prevent buckling during sample expansion.

**Figure 5:**
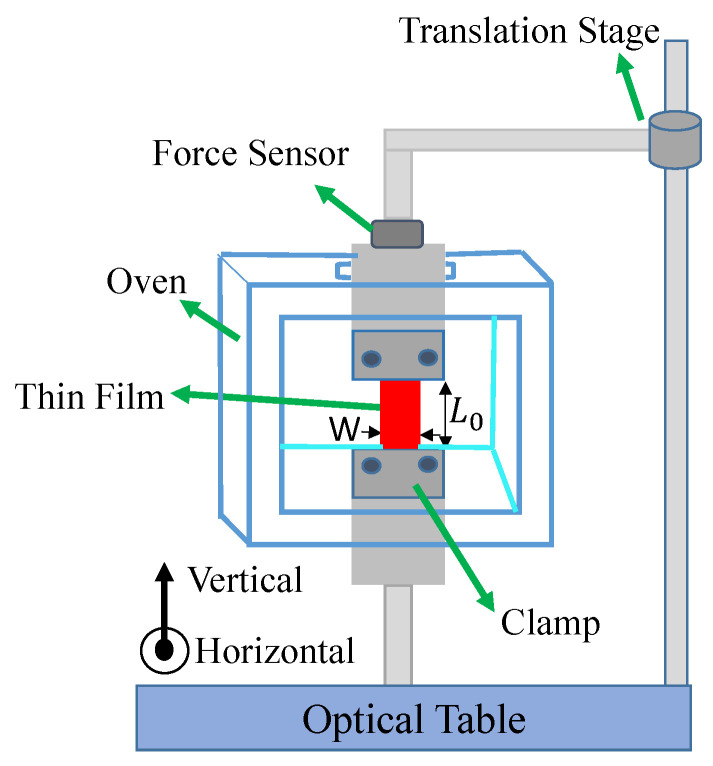
The force measurement setup and sample, which is placed at the intersection of the three beams as diagrammed in Figure 4.

The same setup measures Young’s modulus by translating the upper clamp to stretch the sample. The setup records the strain and force to determine Young’s modulus. The in situ oven enables the temperature-dependent stress to be measured, which can be used to determine the effect of heating on the sample’s properties. Figure 5 shows the force measurement setup used to hold the sample and measure the Young’s modulus and the stress.

### 4.3. Experimental Procedure

The λ=488 nm line of an argon ion laser is used as the light source from which all the other beams are derived. For DR1-doped PMMA samples: 0.5 wt% DR1, $L_{0}=5.3\;\mathrm{mm}$, thickness t=10μm, width w=4.8mm and area $A=tw=4.76\times 10^{-8}\;m^{2}$ in the Young’s modulus experiment and $L_{0}=8.28\;\mathrm{mm}$, thickness t=10μm, width w=4.73mm and area $A=tw=4.73\times 10^{-8}\;m^{2}$ in the photomechanical experiments. For D011-doped PMMA samples: 1.38 wt% DO11, $L_{0}=5.3\;\mathrm{mm}$, thickness t=10μm, width w=4.7mm and area $A=tw=4.74\times 10^{-8}\;m^{2}$ in the Young’s modulus experiment and $L_{0}=8.28\;\mathrm{mm}$, thickness t=10μm, width w=4.6mm and area $A=tw=4.56\times 10^{-8}\;m^{2}$ in the photomechanical experiments.

The experiments seek to determine the parameters required to determine the contributions of angular hole burning and photothermal heating to the photomechanical response κ from temperature-induced stress measurements, Young’s modulus measured at room temperature, and simultaneous pump/probe/stress measurements. We use the data to determine the efficiency of the material in converting light energy to mechanical force. This efficiency provides a metric for comparing materials.

## 5. Results and Discussion

This section brings together all of the pieces, where the theory is applied to the data and the results interpreted.

Section 5.1 starts with introducing two hypotheses based on the observed long-term data. Section 5.1.1 summarizes the parameters determined form the temperature-dependent stress experiment, which will be used to model the photothermal heating contribution. Section 5.1.2 applies the theory of hole burning and photo-stress anisotropy to the polarization-dependent photomechanical response to determine the long-time contributions of the mechanisms. Section 5.2 uses dichroism to determine how the orientational order of the material evolves over long times. The conclusion is that molecular reorientation and orientational hole burning are taking place, but these effects do not couple strongly to the polymer, and so they do not result in an appreciable photomechanical response. Also found are small permanent changes in the material order. Section 5.3 quantifies photomechanical efficiencies by a figure of merit. Section 5.4 separates the amplitudes of the photomechanical response at the two characteristic response times in an effort to associate individual mechanisms with each time scale, using predictions of the photothermal heating theory to determine which timescale corresponds to heating. The results found are partially consistent with expectations, but unexpected results bring up new questions that will require further research to answer.

### 5.1. Photomechanical Response

Comparisons of light-induced stress and absorbance in DR1- and DO11-doped PMMA thin films can be used to deduce the mechanisms of the photomechanical response in photoisomerizable samples. The entire area of the sample is illuminated by the pump in a 15 s on and 15 s off cycle. After multiple repetitions, the intensity is increased and the protocol repeated. Typically, the experiment cycles through fifteen different intensities.

As shown in Figure 2, the photomechanical stress response as a function of time for fixed pump intensity follows a bi-exponential function of the form given by Equations (2) and (3). The data are fit to the model with a time offset of $t_{0}=15\;s$ to determine the time constants and amplitudes.

**Figure 6:**
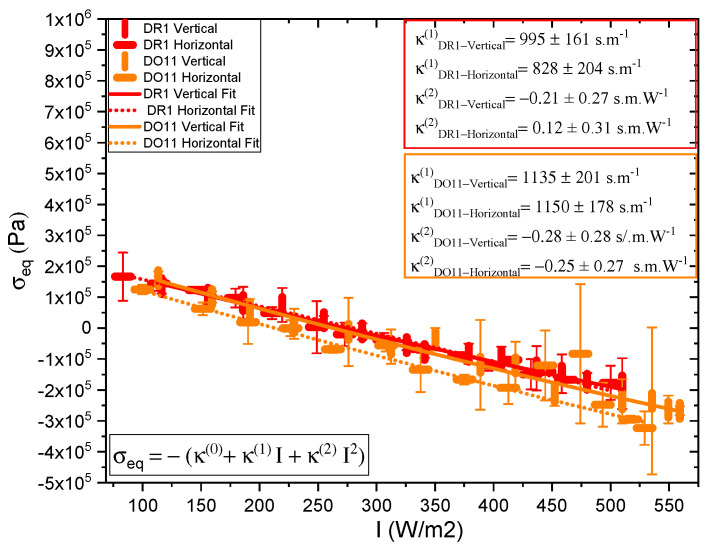
Photomechanical stress response of DR1 and DO11 thin films for horizontal and perpendicular polarization of light as a function of intensity at infinite time, when all processes have come into equilibrium.

One parameter of interest is the long-time stress response $\sigma_{eq}\left(t\to \infty \right)=\sigma_{1}\left(I\right)+\sigma_{2}\left(I\right)$, where $\sigma_{1}\left(I\right)$ and $\sigma_{2}\left(I\right)$ come from the fits to the data to Equations (2) and (3) at each intensity. These are plotted in Figure 6. The long-term stress response of DR1 and DO11 is the same within experimental uncertainties at each intensity. **However, this does not mean that $\sigma_{1}\left(I\right)$ of DR1 is the same as $\sigma_{1}\left(I\right)$ of DO11 and that $\sigma_{2}\left(I\right)$ of DR1 is the same as $\sigma_{2}\left(I\right)$ of DO11, just that the sum $\sigma_{2}\left(I\right)+\sigma_{2}\left(I\right)$ is the same.** As such, the heating contribution to the photomechanical response may be drastically different in the two materials even though their photomechanical response is the same.

The data for each polarization are fitted to a quadratic polynomial which is in the form of Equation (1), and it shows that $\kappa^{\left(1\right)}$I is large compared to $\kappa^{\left(2\right)}I^{2}$ over the data range, so the stress response is linear within fractional deviations of 0.02 and 0.13 for DR1 and 0.05 and 0.12 for DO11 with horizontal and vertical polarizations of light, respectively, where the fractional deviation is given by $\left(\kappa^{\left(2\right)}I^{2}\right)/\left(\kappa^{\left(1\right)}I+\kappa^{\left(2\right)}I^{2}\right)$ at the highest measured intensity.

The linear photomechanical constant is the same within experimental uncertainty for both pump polarizations, i.e., $\kappa_{⊥}^{\left(1\right)}=\kappa_{//}^{\left(1\right)}$, in DR1 and in DO11. This observation leads to two possible hypotheses.

**Hypothesis 1.**  *This observation is as expected in DO11, which has only one conformation and so it should be dominated by photothermal heating alone, a process that is polarization-independent. However, since DR1 is known to photoisomerize [1,4], which leads to angular hole burning [1,4,28,29,30], the data suggest that the light-induced change in the molecular orientational distribution function does not translate into a large enough photomechanical response to be detectable within experimental uncertainties relative to the dominating photothermal heating mechanism. In addition, the magnitudes $\kappa_{⊥}^{\left(1\right)}$ and $\kappa_{//}^{\left(1\right)}$ of DO11 and DR1 agree with each other within experimental uncertainty, strengthening the conclusion that the mechanism of the response is the same in both materials. However, as seen in Figure 6, the horizontal polarization data for DR1 dye is systematically lower than the vertical polarization data and at the edge of experimental uncertainty, allowing for a small contribution from a mechanism that is not found in DO11. This could originate from photoisomerization.*

**Hypothesis 2.**  *Heating and other mechanisms are contributing to the DR1-doped polymers and the magnitude of the heating effect is different in each material so that the individual mechanics contribute differently but in aggregate give the same total response observed in Figure 6. Testing these hypotheses requires that each mechanism be isolated.*

The sections below describe how one isolates each mechanism by controlling individual experimental parameters such as temperature and intensity while monitoring the stress and transmitted probe intensity.

#### 5.1.1. Heating

Thermally induced stress of clamped DR1 and DO11 thin films is studied by placing the samples in an oven with no light applied and measuring the stress as a function of temperature from 291.15 K to 307.15 K over a time span of 3 min. The result is shown in Figure 3. The data are fit to Equation (4) and the fitting parameters are shown in Table 1.

Equation (9) has the parameters *n*, $T_{0}$ and $\sigma_{B}$, which are determined from fits to Equation (4) to temperature-dependent stress data as shown in Figure 3. *T* is the temperature of the sample prior to illumination and τ is the time constant of heating, which can be determined theoretically from the geometry of the material or empirically from the measured stress as a function of time [32]. **The time constant τ can depend on the intensity of the pump beam, which gives $\kappa_{\sigma }^{\left(1\right)}\left(I\right)$ its dependence on intensity.**

The large difference between DR11- and DO11-doped films in Figure 3 implies that, for the same amount of absorbed light energy, the photothermal mechanism in DR1-doped polymers will be larger than in DO11-doped polymers and that the other mechanisms, when added to heating, makes the total response similar in the two materials. This supports the second hypothesis.

Determining the cause for this large difference is beyond the scope of this paper but is a worthy goal for future studies. There are potentially multiple causes for this difference including the number of internal degrees of freedom added by the dopant (isomerization in DR1 and tautomerization in DO11), which can be modeled with statistical mechanical approaches, the efficiency of heat transfer to the polymer relative to the other competing processes such as molecular shape changes and plasticization due to the dyes. Combinations of these processes working together could magnify the effects of each.

#### 5.1.2. Hole Burning and Photo-Stress Anisotropy

Table 2 summarizes the parameters determined from fits to the data shown in Figure 6 for thin films. For comparison, also shown are the values from the literature for dye-doped fibers [2]. The effect of dye-doping and annealing of the photomechanical effect in fibers has also been studied [33]. The positive sign of $\kappa^{\left(\mathrm{heat}\right)}$ in both materials and in both thin films and fibers indicates that photothermal heating drives expansion. The ratio of the hole burning to the heating response, *R*, vanishes within experimental uncertainty, confirming that photothermal heating dominates in thin films and fibers. In DO11 $\kappa_{//}^{\left(\mathrm{hole}\right)}$ and $\kappa_{⊥}^{\left(\mathrm{hole}\right)}$ are expected to be zero, because DO11 is nonisomerizable, and the results show this to be true with a high degree of confidence. So, we conclude that angular hole burning and molecular reorientation are absent in DO11. DR1 shows a larger contribution from angular hole burning, and the signs of the coefficients are what is expected but vanish within experimental uncertainty. The sign of $\kappa_{//}^{\left(\mathrm{hole}\right)}$ indicates that the length of the sample along the light’s polarization decreases and the positive sign of $\kappa_{⊥}^{\left(\mathrm{hole}\right)}$ shows that the length of the sample increases along its long axis when it is illuminated by light perpendicular to it, refs. [1,2], as would be expected. But again, the values are statistically insignificant, so angular reorientation and hole burning are negligible.

**Figure 7:**
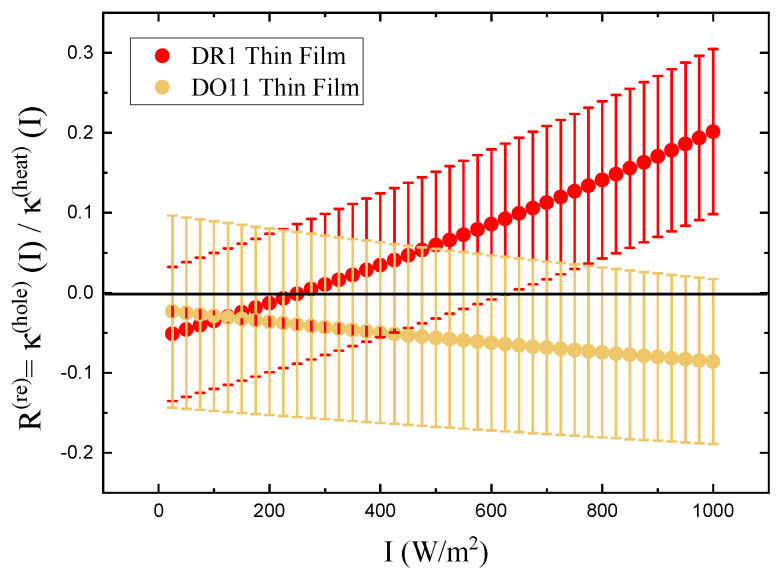
The ratio of the photomechanical stress due to angular hole burning and heating.

To make sure that the response is linear and we are in the low-intensity regime, the ratio *R* as a function of intensity is also calculated for both samples. *R* is found using Equation (26). Figure 7 shows a plot of the relative reorientational fraction *R* as a function of intensity, where we have evaluated $\kappa_{//}^{\left(1\right)}+\kappa_{//}^{\left(2\right)}I$ and $\kappa_{⊥}^{\left(1\right)}+\kappa_{⊥}^{\left(2\right)}I$ in Equation (26) using the values of $\kappa_{⊥}^{\left(1\right)}$, $\kappa_{⊥}^{\left(2\right)}$, $\kappa_{//}^{\left(1\right)}$, and $\kappa_{//}^{\left(2\right)}$ determined from the slopes in Figure 6 to obtain the points at each intensity *I*. The error bars are propagated from the measured uncertainties in $\kappa_{⊥}^{\left(1\right)}$, $\kappa_{⊥}^{\left(2\right)}$, $\kappa_{//}^{\left(1\right)}$, and $\kappa_{//}^{\left(2\right)}$. The ratio varies more over the intensity range measured for DR1 than for DO11. The ratio *R* for DR1 appears to change sign at low intensity, but this sign change is in the noise given the uncertainties. The increase in *R* for DR1 is statistically significant, suggesting that hole burning becomes a significant fraction of the response mechanisms at the highest intensities.

While the behavior of the thin films and fibers is similar, the linear photomechanical constants are a factor of three to four times larger in the thin films.

### 5.2. Evolution of Molecular Orientation

Section 5.1 describes how the photomechanical effect depends on the various mechanisms. Here we focus on measurements that directly determine the orientational order of the dopant molecules in the PMMA polymer host, which can be correlated with the photomechanical response to assess if the orientational order couples mechanically to the polymer and that it contributes to photomechanical actuation.

The transmitted and incident intensities are recorded using a power meter with and without a sample. From these, the optical absorption coefficient of the sample is determined for the two orthogonal polarizations using the Beer–Lambert law [1,2]

(28) $$\begin{array}{c} I=I_{0}e^{-\alpha d}, \end{array}$$

where

(29) $$\begin{array}{c} \alpha_{//}=-\frac{1}{d}\ln\left(\frac{I_{//}}{I_{0}}\right), \end{array}$$

and

(30) $$\begin{array}{c} \alpha_{⊥}=-\frac{1}{d}\ln\left(\frac{I_{⊥}}{I_{0}}\right). \end{array}$$

**Figure 8:**
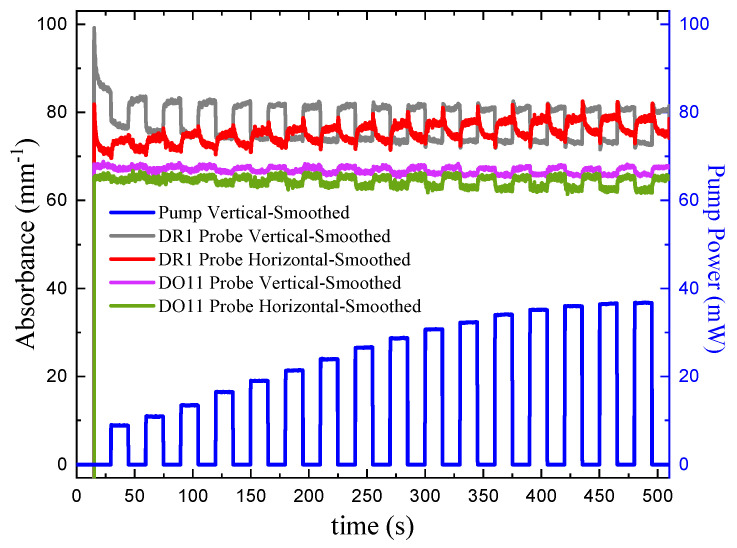

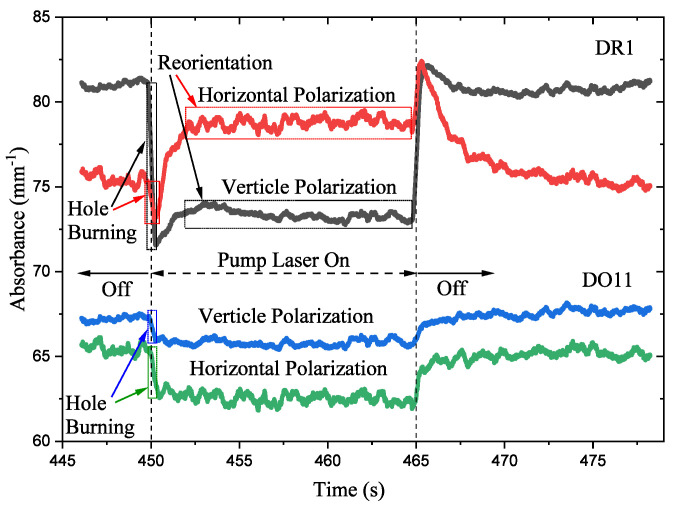
(**top**) Absorbance parallel and perpendicular to the pump beam polarizations in DR1- and DO11-doped PMMA thin films as a function of time with increasing pump intensity. (**bottom**) A close-up of the data for one on–off cycle showing a fast process associated with angular hole burning and two slow processes, which includes molecular reorientation.

$\alpha_{//}$ and $\alpha_{⊥}$ are the absorbance parallel and perpendicular to the polarization of the pump beam, respectively. Figure 8 (top) shows the absorbance for vertically and horizontally polarized probe beams in DR1- and DO11-doped PMMA thin films. Figure 8 (bottom) shows a close-up view of one cycle in a region where transients from the probe lasers being turned on have subsided. For DR1, the vertically polarized pump induces a fast increase in the intensities of both probe polarizations as shown by the decreased absorbance. At later times with the pump on, the intensity of the horizontally polarized probe beam decreases (increased absorbance), while the vertically polarized probe beam shows little change.

This fast behavior can be understood as follows. Irradiating DR1 molecules with vertically polarized light preferentially excites the vertically oriented chromatophores. In the argument here, we assume that DR1 absorbs less light in its excited state and the molecules are one-dimensional, which has been shown to be a good approximation [34]. The probability of exciting a chromophore that is oriented an angle θ relative to the pump polarization is proportional to $\cos^{2}\theta$, making it most probably to excite vertically oriented molecules at θ=0 and improbably in the horizontal direction, where θ=π/2. The probability of a molecule absorbing vertically polarized probe light is proportional to $\cos^{2}\theta$, making the probability of absorbing vertically polarized probe light in the presence of the vertically polarized pump beam is proportional to $\cos^{4}\theta$. Similarly, since the probability of absorbing horizontally polarized light is proportional to $\sin^{2}\theta$, the probability of absorbing horizontally polarized probe light in the presence of a vertically polarized pump is proportional to $\sin^{2}\theta \cos^{2}\theta$.

Finally, assuming that the sample is isotropic, which should be based on the material’s processing conditions, the ratio of the change in absorbance for the two polarizations for an initially isotropic sample is given by the orientational average ratio

(31) $$\begin{array}{c} \frac{\alpha_{⊥}}{\alpha_{//}}=\frac{\langle \sin^{2}\theta \cos^{2}\theta \rangle }{\langle \cos^{4}\theta \rangle }=\frac{2}{3}. \end{array}$$

Equation (31) implies that the drop in the vertically polarized probe is greater than the horizontal one and that they both should drop [1,2]. Figure 8 (bottom) shows this to be true for DR1. DO11, on the other hand, also shows a drop in the absorbance for both polarizations, but the one-dimensional approximation does not hold as well for DO11. In both cases, we see a drop in the absorbance due to the pump light exciting the molecules. This effect is labeled “hole burning” because the orientational distribution function takes on a shape like the dimple in an apple. Note that the probe beam intensities are much lower than the pump, so their affect on the excited populations should be minimal.

Another fast process is photothermal heating, which increases the temperature of the material as the absorbed light is converted to heat. Since the trans isomer is of lower energy than the cis isomer, the trans population drops and the cis population increases at higher temperatures, leading to an isotropic decrease in the absorbance of light by virtue of the cis molecule’s slow absorption cross-section. Thus isotropic heating will lower the absorbance of both polarizations equally. The fast component will thus be a combination of both heating and hole burning.

At longer time scales, molecules that undergo photoisomerization will reorient into the horizontal direction, as we would expect; when molecules aligned along the light’s polarization turn into the smaller cis state, they reorient randomly due to diffusion and on average end up in the perpendicular orientation once they decay back to the trans state [2,4]. This decreases the vertical absorbance because fewer molecules are aligned with the pump beam and increases the horizontal absorbance where there are more molecules. The horizontal probe sees an increase in absorbance as we would expect. Similarly, the long-time vertical probe sees a decrease in the absorbance, as expected. At an intermediate time between the fast hole-burning process and the slow molecular reorientation process, another feature is observed where both probe polarizations show an increase in absorbance. The region’s time scale includes the photothermal heating mechanism, which can change the relative populations between the cis and trans isomers. Separating the mechanisms to this level of detail is beyond the scope of this work, but it is worth mentioning that the three mechanisms can couple to produce complex behavior, which is difficult to model.

DO11 thin films show a decrease in absorption for both polarizations in response to the pump and an increase when the pump is turned off. The DO11 molecule is known to transition from the keto to the enol state when excited by a photon. Optical absorption of the enol state is weaker, so the absorbance decreases when the pump is on. The DO11 molecule relaxes back to the keto state when the pump is turned off [26,35], leading to an increase in the absorbance.

In both DR1 and DO11 molecules, the time dependence of the absorbance when light is turned off is the reverse of the process when the light is turned on. In the case of DR1, where there are multiple exponential processes that contribute amplitudes of opposite signs, the process is left–right anti-asymmetric, while the DO11 process is left–right symmetric. Note that for both cases, flipping the pump-off absorbance data about the horizontal axis and shifting them by 15 s gives the same qualitative shape.

The difference in the absorbance measured by the two orthogonal probe lasers can be used to find the axial orientational order parameter $\langle P_{2}\rangle$. For molecules that are approximately one-dimensional, the order parameter is given by [26,30]

(32) $$\begin{array}{c} \langle P_{2}\rangle =\frac{\alpha_{//}-\alpha_{⊥}}{\alpha_{//}+2\alpha_{⊥}}. \end{array}$$

The order parameter $\langle P_{2}\rangle =1$ if the long axes of the molecules are fully aligned and $\langle P_{2}\rangle =0$ when they are randomly aligned. The order parameter can be negative when the molecules lie flat in the plane perpendicular to the symmetry axis.

**Figure 9:**
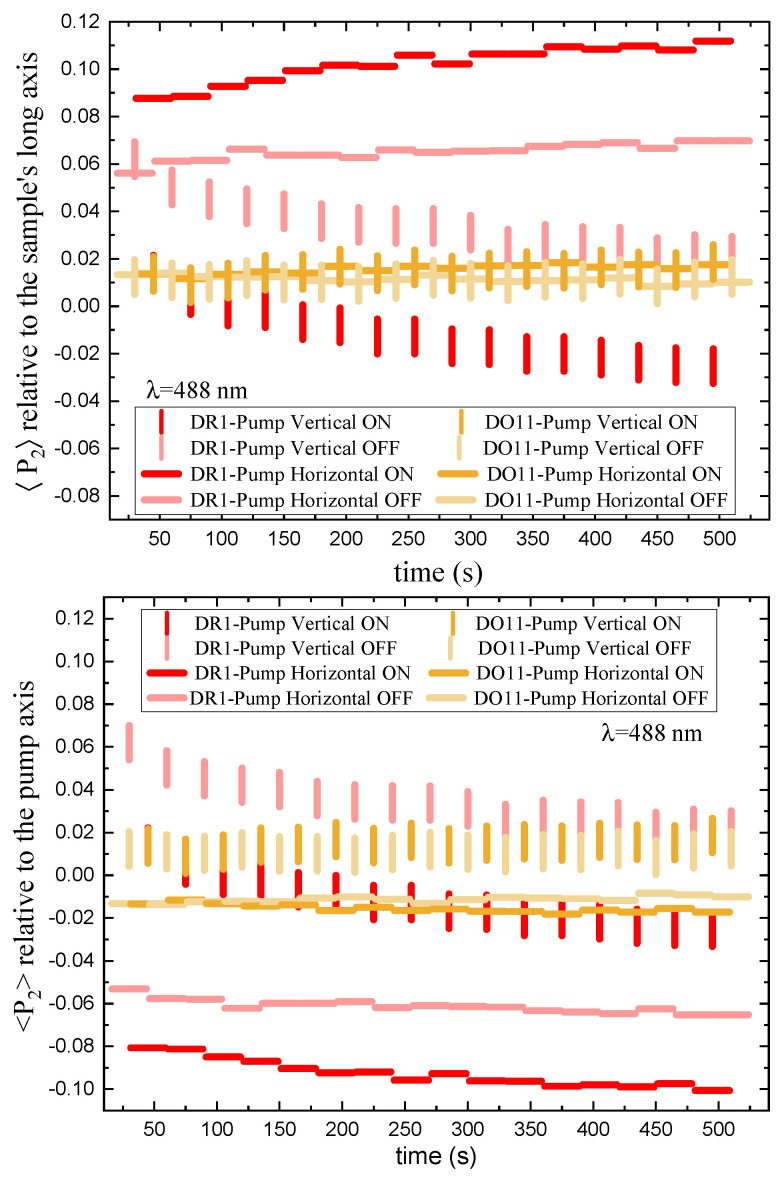
Measured order parameter of DR1- and DO11-doped PMMA thin films as a function of time. Time is a proxy for intensity as shown in the pump power plots on the bottom part of the top graph of Figure 8. (**top**) Order parameter relative to the sample’s long axis and (**bottom**) order parameter relative to the pump beam’s polarization axis.

The order parameter relative to the sample’s long axis, mounted vertically, as a function of time and intensity is shown in Figure 9 for vertically and horizontally polarized pump light. The top plot references the order parameter to the long axis of the sample while the bottom plot is relative to the pump axis.

Order parameter relative to long axis of sample: We first consider the top plot of Figure 9, which references the order parameter to the long axis of the thin-film sample. The long axis is always mounted vertically. Turning the pump on and off for both polarizations does not affect the order parameter in DO11—as expected when photo-isomerization is absent. In contrast, for DR1, a *vertically polarized pump* decreases the order parameter along the film’s long axis (vertical) and turning the pump off results in an increase in this order parameter back to its pre-pumped state. A *horizontally polarized pump* increases the order parameter, which decreases reversibly when the pump is turned off. This behavior is expected if the pump light that is polarized along the trans molecule’s long axis excites the cis state, making a “hole” along the light’s polarization direction by depleting those trans molecules. Subsequently, the number of molecules in the perpendicular orientation increases as the cis molecules relax back to the trans state and take on random orientation but leave a net depletion along the pump’s polarization.

Order parameter relative to pump polarization axis: The bottom part of Figure 9 shows the order parameter relative to the pump’s polarization axis. For an isotropic sample, the order parameter in a dark sample should vanish and the order parameter in the presence of pump light should be the same for both polarizations if their intensities are the same. The data show that the order parameters are not the same for the two polarizations, so the samples are not isotropic. There are several possible sources of this anisotropy, including the fact that the samples are slightly stretched in the vertical direction when mounted in the sample holder. This is consistent with the observation that a vertical pump polarization has a slight positive order parameter, while the horizontal one has a negative order parameter. In both cases, the presence of the pump leads to a decrease in the order parameter, as expected for molecular reorientation.

**Figure 10:**
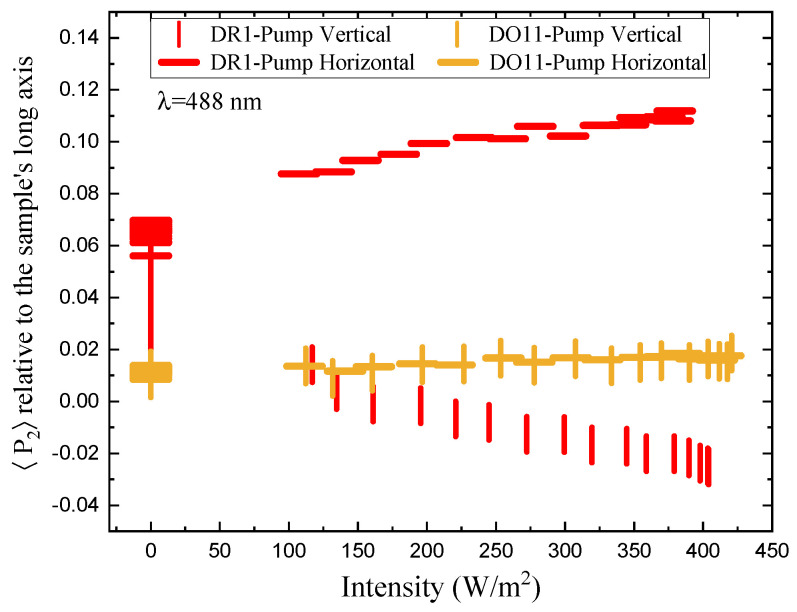

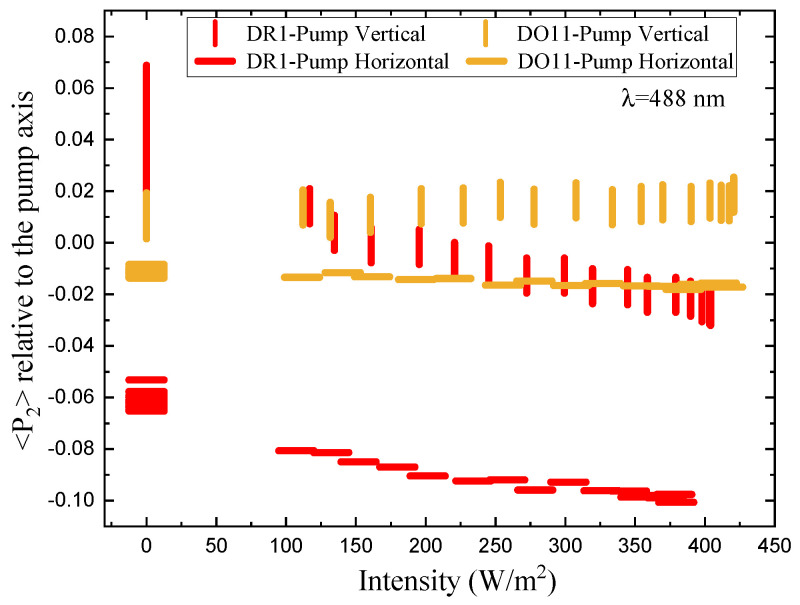
Order parameter of DR1- and DO11-doped PMMA thin films as a function of intensity. (**top**) Order parameter relative to the sample’s long axis and (**bottom**) order parameter relative to the pump’s polarization axis for vertically (vertical lines) and horizontally polarized (horizontal lines) pump. The data markers at I=0 include the initial measurement before the start of the experiment, as well as all the order parameters measured when the pump light is blocked prior to increasing intensity (the I=0 wells of the pump power are plotted in the upper portion of Figure 8).

Figure 10 shows the same data as in Figure 9, but as a function of pump power. Because the samples are slightly anisotropic, the behavior is made clearer by considering the change in the order parameter relative to the dark state. This change in the order parameter $\Delta \langle P_{2}\rangle$ is given by

(33) $$\begin{array}{c} \Delta \langle P_{2}\rangle \left(I\right)={\langle P_{2}\rangle }_{on}\left(I\right)-{\langle P_{2}\rangle }_{off}\left(0\right), \end{array}$$

where ${\langle P_{2}\rangle }_{on}\left(I\right)$ is the order parameter when the pump intensity is *I* and ${\langle P_{2}\rangle }_{off}\left(0\right)$ is the order parameter of the material prior to the start of the experiment. We call ${\langle P_{2}\rangle }_{on}\left(I\right)$ the total order parameter change because it includes the change in order parameter due to the instantaneous pump power, as well as the accumulated order parameter from all past exposure due to the fact that the dye molecules have not had the chance to relax to the isotropic state during the 14 s period that the pump light is turned off. The low levels of probe light might also add to the cumulative effect. The values of ${\langle P_{2}\rangle }_{on}\left(I\right)$ at I=0 include all the values determined when the pump light is off between measurements where the intensity is changed. If there were no cumulative effects of time as the pump intensity is increased, there would be only one point for each polarization and each material.

**Figure 11:**
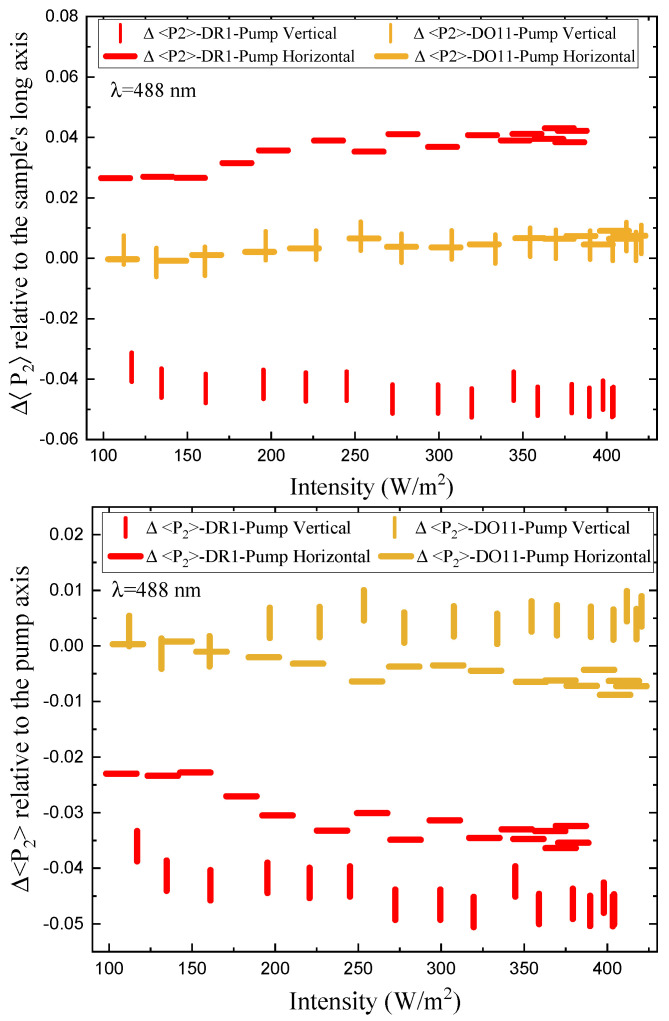
Change in the order parameter in DR1- and DO11-doped PMMA thin films relative to the order parameter of the pristine sample as a function of the pump intensity. This includes the order parameter change induced by the light and the accumulated change in the order parameter from past exposures. (**top**) Order parameter relative to the long axis of the sample and (**bottom**) order parameter relative to the pump beam’s polarization axis.

Figure 11 shows the order parameter change $\Delta \langle P_{2}\rangle$ as a function of absorbed intensity relative to the sample’s long axis (top) and the pump axis (bottom). For DO11-doped PMMA, the order parameter referenced to the long axis of the sample (top plot) shows no statistically significant change as a function of intensity, while DR1-doped PMMA shows a dramatic effect with the horizontal pump inducing an increase in order parameter and the vertical pump inducing a decrease. The decrease in order parameter for the vertical pump is of slightly larger magnitude than the increase induced by the horizontal pump. These observations are consistent with molecules reorientating away from the light’s polarization and ending up in the plane perpendicular to the pump.

The order parameter referenced to the pump axis (bottom plot) shows a larger difference between the two pump polarizations for DO11 and a similar difference for DR1. But, the trend is the same; the order parameter drops in the direction of the pump polarization axis. The observation of a pump polarization-dependent change in the parameter suggests that the sample might be initially anisotropic. The change in the order parameter $\Delta \langle P_{2}\rangle$ appears to saturate at higher intensities, which one would expect for a fixed population of molecules.

Next, we seek to understand the source of the anisotropy. The two obvious candidates are (1) anisotropy due to the slight stretching of the sample along its long axis from mounting strain and (2) long-time molecular reorientation that builds over time if the dyes in the polymer do not have enough time to relax over the 15 s dark cycle when the pump is off. There may also be an effect due to the excitation of molecules due to the ever-present weak probe beams, whose small effects may accumulate over time.

First, we need to determine the change in order parameter due to the current pump cycle. This is best characterized by the short-time order parameter change, defined as the fractional change in the order parameter, which is normalized to the order parameter just before the pump is turned on during each cycle, ${\langle P_{2}\rangle }_{off}$(n), given by

(34) $$\begin{array}{c} {\frac{\Delta \langle P_{2}\rangle }{\langle P_{2}\rangle }}^{\left(fast\right)}\left(n\right)=\frac{{\langle P_{2}\rangle }_{on}\left(n\right)-{\langle P_{2}\rangle }_{off}\left(n\right)}{{\langle P_{2}\rangle }_{off}\left(n\right)}, \end{array}$$

where *n* labels the cycle number.

**Figure 12:**
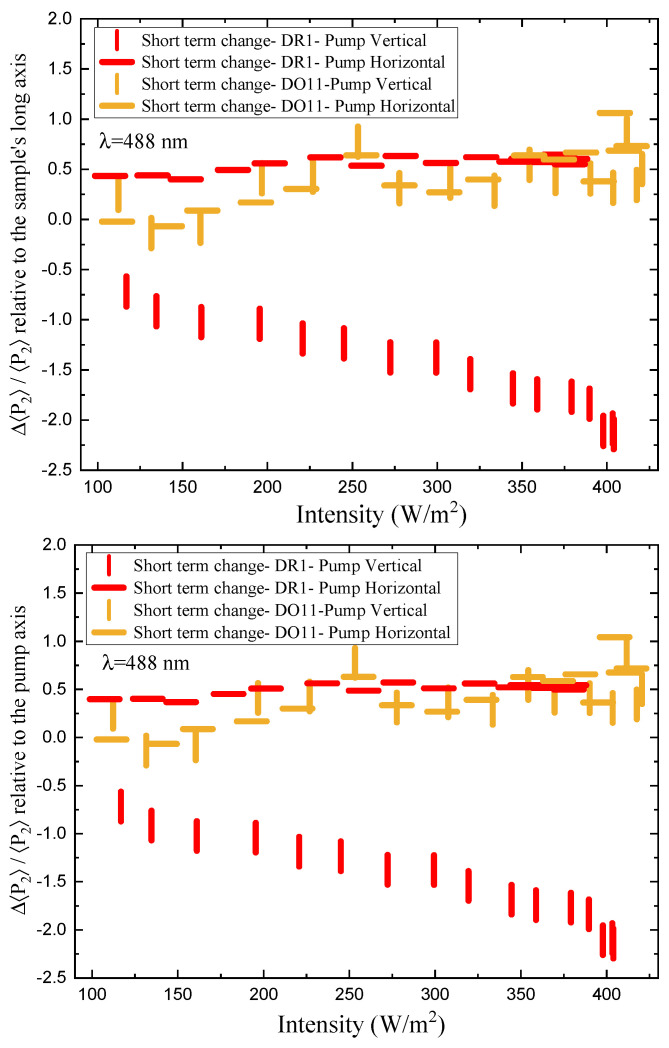
Fractional change in the order parameter relative to the order parameter just prior to the pump being turned on as a function of pump intensity in DR1- and DO11-doped PMMA thin films. (**top**) Fractional order parameter change relative to the sample’s long axis and (**bottom**) relative to the pump axis for both vertically and horizontally polarized pump light.

Figure 12 shows the short-time fractional order parameter change as a function of intensity relative to the sample’s long axis (top) and relative to the pump’s polarization axis for both vertically and horizontally polarized pumps. DO11 shows the same fractional order parameter change for both pump polarizations, which is consistent with the absence of molecular reorientation. DR1, on the other hand, is consistent with molecular reorientation in that the order parameter change relative to the long axis for a vertically polarized pump is greater than for a horizontally polarized pump and of opposite sign. In the low-intensity regime, the ratio is about −2/3, as predicted by Equation (31). The ratio becomes smaller at higher intensities, which is an indication that the sample is changing over time due to pump cycling and the effects of the probe beams. This does not preclude an intensity-dependent effect that originates in nonlinearities in the underlying mechanisms. Note that extrapolating the plots to zero intensity suggests clustering around a vanishing fractional order parameter change.

The long-term effects of the light on the sample can be assessed by comparing its properties at a given cycle with the sample prior to the first cycle. To this end, we define the long-term fractional change in the order parameter in the $n^{\mathrm{th}}$ cycle by referencing it to the order parameter of the sample at the start of the measurement. We start by determining the fractional change in the order parameter when the pump is off after the $n^{\mathrm{th}}$ cycle, given by

(35) $$\begin{array}{c} {\frac{\Delta \langle P_{2}\rangle }{\langle P_{2}\rangle }}^{\left(slow,dark\right)}\left(n\right)=\frac{{\langle P_{2}\rangle }_{off}\left(n\right)-{\langle P_{2}\rangle }_{off}\left(first\right)}{{\langle P_{2}\rangle }_{off}\left(first\right)}. \end{array}$$

This will determine the change in the dark state of the sample in the presence of only the weak probe lasers. Doing the same for when the pump is on during the $n^{\mathrm{th}}$ cycle, we obtain

(36) $$\begin{array}{c} {\frac{\Delta \langle P_{2}\rangle }{\langle P_{2}\rangle }}^{\left(slow,pump\right)}\left(n\right)=\frac{{\langle P_{2}\rangle }_{on}\left(n\right)-{\langle P_{2}\rangle }_{off}\left(first\right)}{{\langle P_{2}\rangle }_{off}\left(first\right)}. \end{array}$$

**Figure 13:**
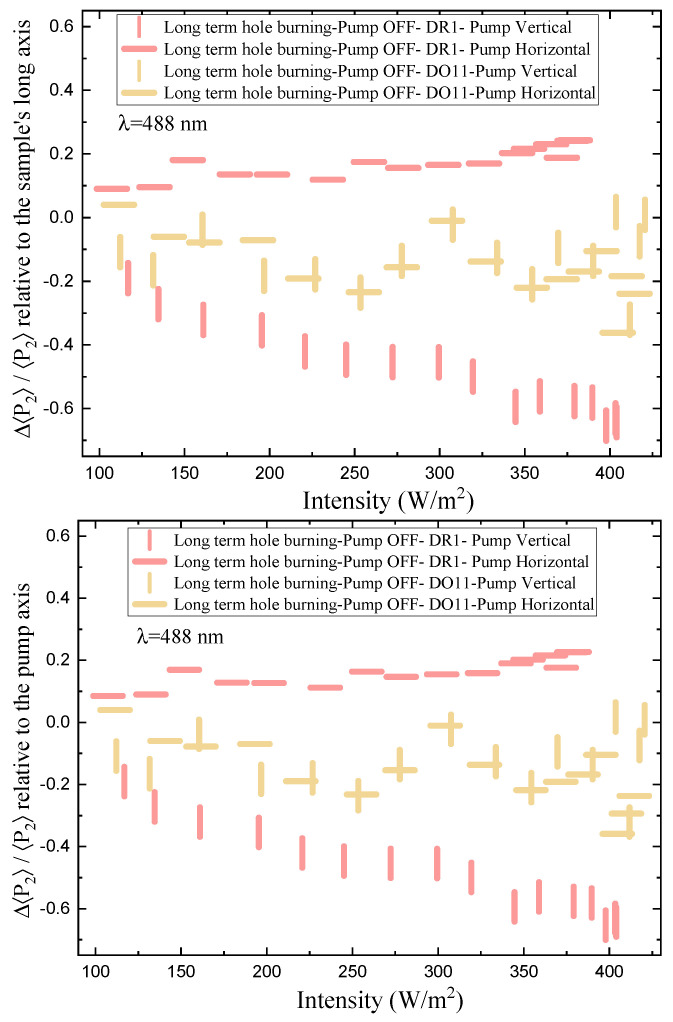
Long-term time evolution of the fractional order parameter change *when the pump is off* relative to the order parameter of the pristine sample as a function of the intensity of the pump prior to the initial measurement at t=0 in DR1- and DO11-doped PMMA thin films. (**top**) Fractional order parameter change relative to the sample’s long axis and (**bottom**) relative to the pump axis for both vertically and horizontally polarized pump light.

**Figure 14:**
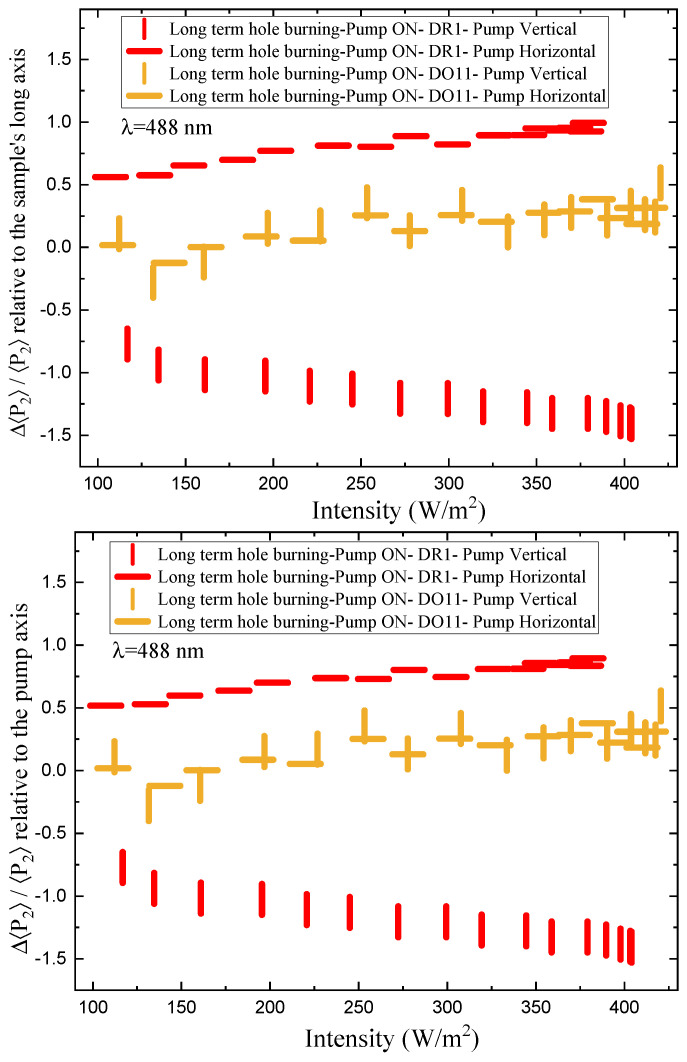
Long-term time evolution of the fractional order parameter change as a function of the pump intensity in DR1- and DO11-doped PMMA thin films. (**top**) Fractional order parameter change relative to the sample’s long axis and (**bottom**) relative to the pump axis for both vertically and horizontally polarized pump light.

The long-term fractional change in the order parameter as a function of intensity when the pump is off and the pump is on relative to the pristine sample are shown in Figure 13 and Figure 14. The data clearly show that the dark state of the material (when pump is off, but the probes are on), over the long-term span of the experiment, evolves over time. The fractional change in the vertically referenced order parameter in DR1 decreases over time (and with increased pump intensity). This change is most likely a combination of the sample’s properties changing over time under the influence of the pump and probe beams, as well as the increased pump intensities, which might produce a longer-lasting change in the material properties. Similarly, when the pump is on, the vertically referenced fractional order parameters decreases over time, but by a greater factor than the dark state. This is attributable to the fact that in the pumped state, the fractional change in the order parameter is influenced by both the long-term changes in the sample and the short-term effects of the pump.

Consider that the fast response shows an increase in the fractional order parameter change over time/intensity from about −0.75 to −2.25, the slow dark state changes from −0.3 to −0.65 and the slow pumped state changes from −0.75 to −1.3. At the lowest intensity and earliest times, there is already evidence of the dark state being anisotropic. At the highest intensity, the fast response shows the largest fractional change in the order parameter, while the long-term response is somewhat lower, but by a statistically significant amount. The long-term change in the fractional order parameter in the dark state is greater than the initial value but still significantly smaller than the pumped state. Thus, the changes in the sample are enough to have a statistically significant effect on the measurements of the pumped state at high intensity, though not a large difference. Changes in the material from long-term light exposure might explain most of the small variations in DO11-doped PMMA thin films. However, the fractional change in the order parameter in DO11 is always similar for both pump polarizations, confirming that angular hole burning and reorientation are insubstantial.

### 5.3. Photomechanical Efficiency

**Figure 15:**
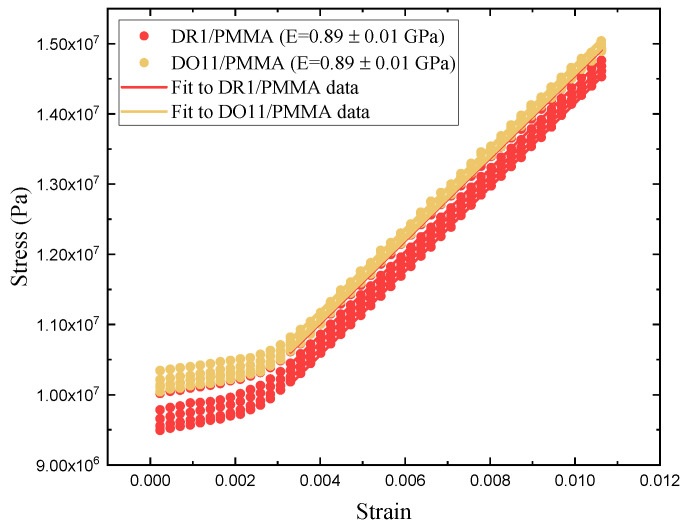
The measured stress versus strain in DR1- and DO11-doped PMMA thin films at room temperature. The plot shows 5 separate runs for each material. The initial slope below a strain of 0.003 is due to system backlash. Young’s modulus is determined from the slope of the average of the 5 runs above a strain of 0.004.

The photomechanical efficiency is given by Equation (27). It requires a measurement of both the photomechanical response and Young’s modulus. The photomechanical response is described above. Here, we determine Young’s modulus of DR1 and DO11 at room temperature by pulling on one end of the sample with a stepper motor running at constant speed while measuring the stress. For each sample, the experiment is repeated five times and the average of the five runs is used to decrease experimental uncertainties. Figure 15 shows the five runs for each sample. The initial flat portion up to a strain of 0.002 is due to backlash in the stepper motor and translation stage used to strain the thin film and also due to the fact that the sample is not rigid and so it is initially in a bucked state when first mounted. At each cycle, the stepper motor brings the thin film back to the same point. Young’s modulus is determined from the slope above a strain of 0.004 where the sample is stretched beyond the buckling point along its long axis. Both DR1- and DO11-doped PMMA give a Young’s modulus of 0.89 ± 0.01 GPa. The stress for all measurements is well below the yield point and far below the stress required to fracture the polymer.

Table 3 summarizes the thin-film results, including the efficiency in converting light energy to mechanical work using the figure of merit. For comparison, results from the literature of DR1-doped PMMA fibers are also presented [2]. For each sample, the figure of merit (FOM) is the same within experimental uncertainty for each of the two pump polarizations in the two dye-doped PMMA samples. Similarly, the thin-film figure of merit is the same within experimental uncertainty for the two materials at the same polarization. One must keep in mind that these are 1-σ error bars.

However, there is a slight statistically significant difference between the two polarizations in DR1. This could be due to the small contribution of reorientation, which is of the same order of magnitude as the error bars. Similarly, the difference between the two different materials is different at the edge of the error bars for the horizontally polarized pump light, where we expect the effects of molecular reorientation to be the largest. But, given that these effects are at the edge of the error band, these differences are insignificant but of the correct polarization-dependence to be due to molecular reorientation.

The FOM for DR1-doped PMMA fibers is about two orders of magnitude lower than for thin films. The FOM is even lower when the fibers are annealed, as described in the literature [33]. Furthermore, the Young’s modulus in the fiber geometry is more than a factor of three larger than in the thin-film geometry. The advantage of the thin films over fibers originates in both parameters; the smaller Young’s modulus provides less material resistance to the photomechanical force which is leveraged by the larger photomechanical constant. The larger photomechanical constant may be due to more efficient heating due to the lower heat capacity of the thin film and the uniform illumination. The lower Young’s modulus of the thin film might be due to surface effects, where the skin—of the lower modulus—contributes more to the response because it contains a larger fraction of the total sample volume. The question of volume versus surface effects is an interesting one which could be studied characterizing samples of the same length but varying cross-sectional aspect ratio.

### 5.4. Photomechanical Time Response

**Figure A1:**
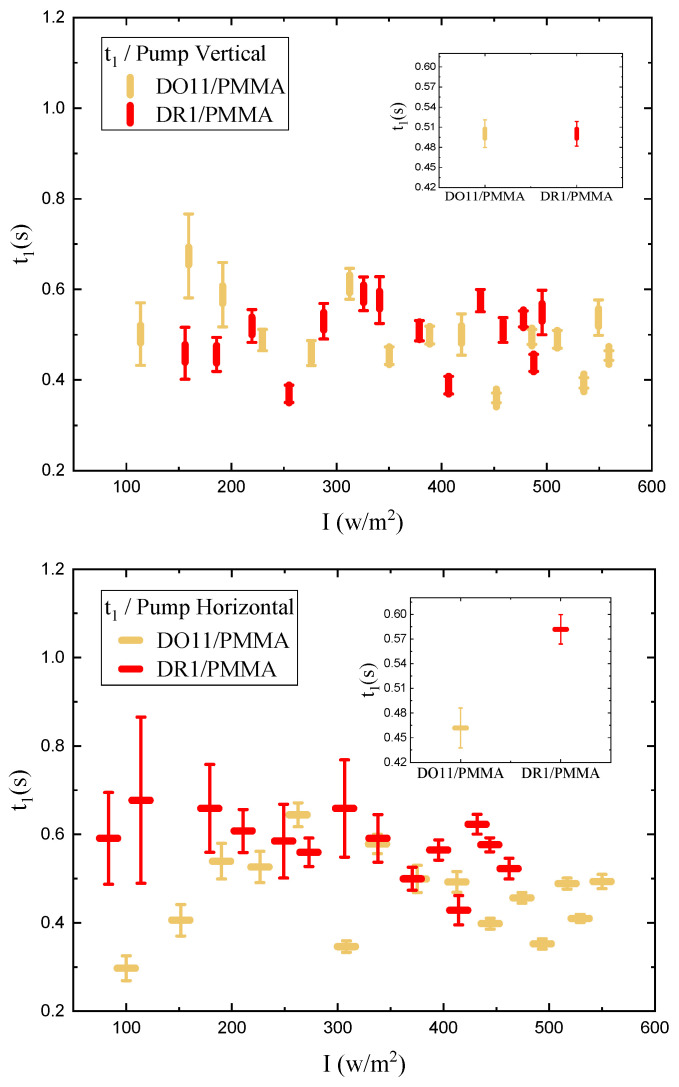
The fast time constant $t_{1}$ of the photomechanical response for DR1- and DO11-doped PMMA thin films as a function of intensity. The inset shows the average time constant over the whole intensity range for each material. (**top**) Vertically and (**bottom**) horizontally polarized pump.

**Figure A2:**
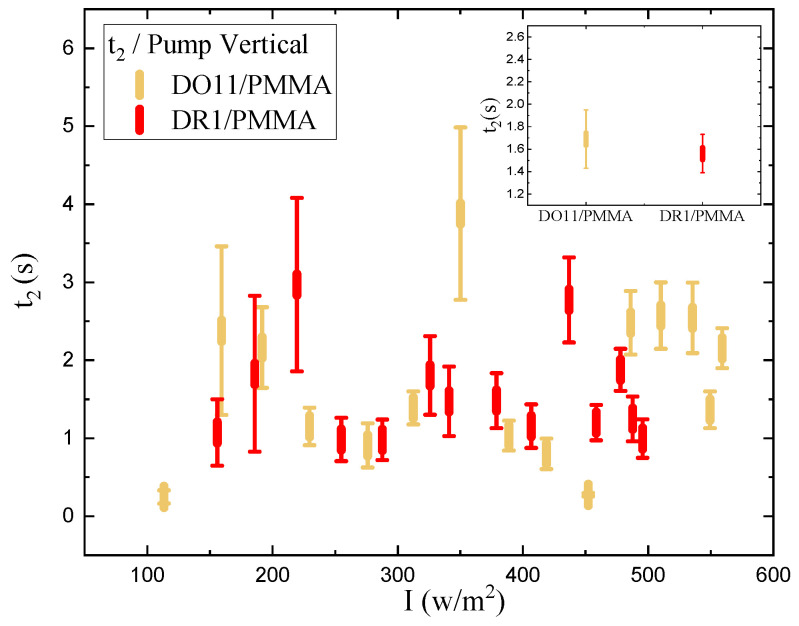

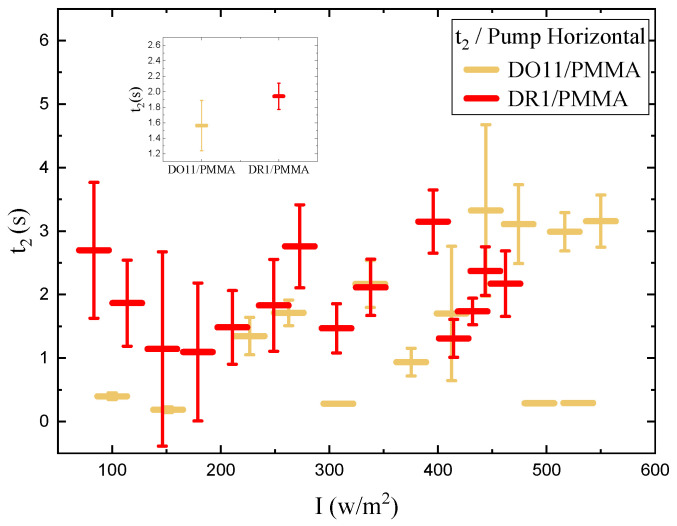
The slow time constant $t_{2}$ of the photomechanical response for DR1- and DO11-doped PMMA thin films as a function of intensity. The inset shows the average time constant over the whole intensity range for each material. (**top**) Vertically and (**bottom**) horizontally polarized pump.

**Figure A3:**
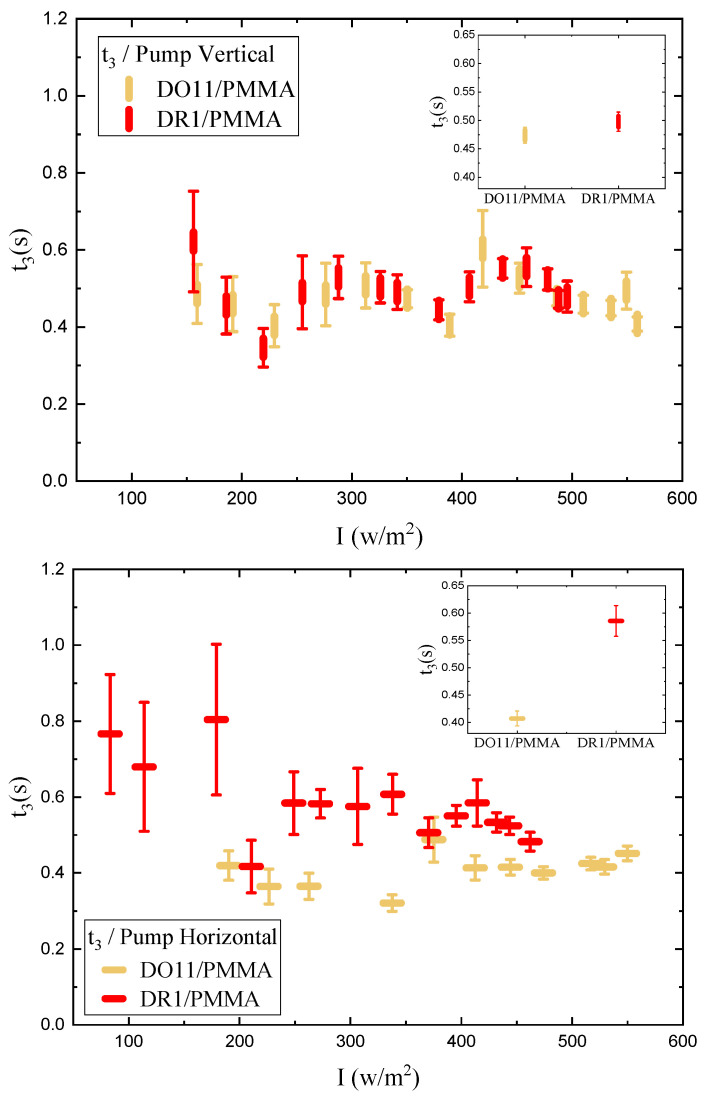
The fast relaxation time constant $t_{3}$ for DR1- and DO11-doped PMMA thin films as a function of intensity. The inset shows the average time constant over the whole intensity range for each material. (**top**) Vertically and (**bottom**) horizontally polarized pump.

**Figure A4:**
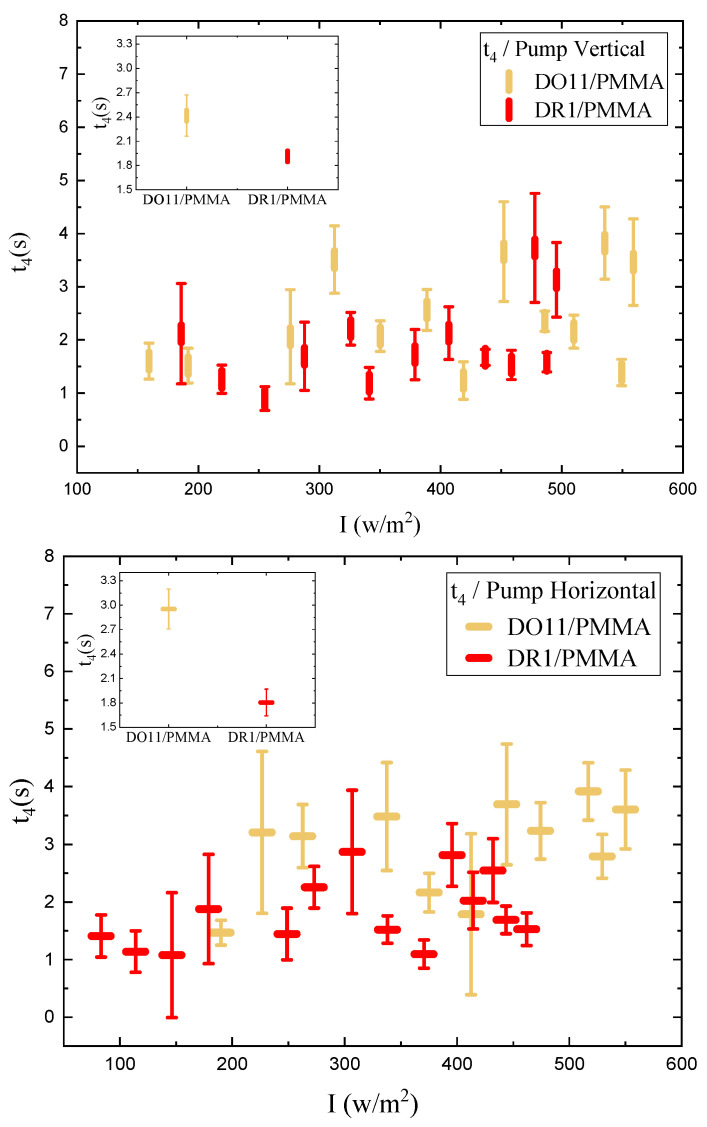
The slow relaxation time constant $t_{4}$ for DR1- and DO11-doped PMMA thin films as a function of intensity. The inset shows the average time constant over the whole intensity range for each material. (**top**) Vertically and (**bottom**) horizontally polarized pump.

We determine the photomechanical response times by fitting the time-dependent stress as shown in Figure 2 to a double exponential when the pump is on and a separate double exponential when the pump is off. As a function of intensity, these fits give the intensity dependence of the magnitude and characteristic photomechanical time constants. Figure A1 and Figure A2 show the time constants for the photomechanical response, while Figure A3 and Figure A4 show the relaxation time constants when the light is turned off for both DR1- and DO11-doped PMMA for both pump polarizations. The insets show the average time constant over the full intensity range for each material at each polarization.

While the fluctuations in the data exceed the error bar range, there is no obvious trend in the intensity dependence. The average of each run for a given sample and pump polarization shows that both the photomechanical response and its relaxation have two characteristic time constants that are about the same for each material. The fast time constant is about 0.5 s for both materials and for both the photomechanical response and the relaxation process. The longer time-scale process shows a different time constant for the pump on and off and range from about 1.5 s for pumped response to 3 s during relaxation.

In contrast, fibers from previous studies display a single time constant for both decay and recovery between about 0.7 s and 1.0 s [2]. This suggests that the underlying processes in the two sample geometries may be different, which might originate in the surface skin effect, where in thin films, the skin is a much larger fraction of the total volume than in fibers. As such, the thin film measurements reported here most likely are probing a regime where the skin contributes a large fraction of the total response, thus showing two unique processes, while the fiber measurements are mostly probing bulk effects.

**Figure 16:**
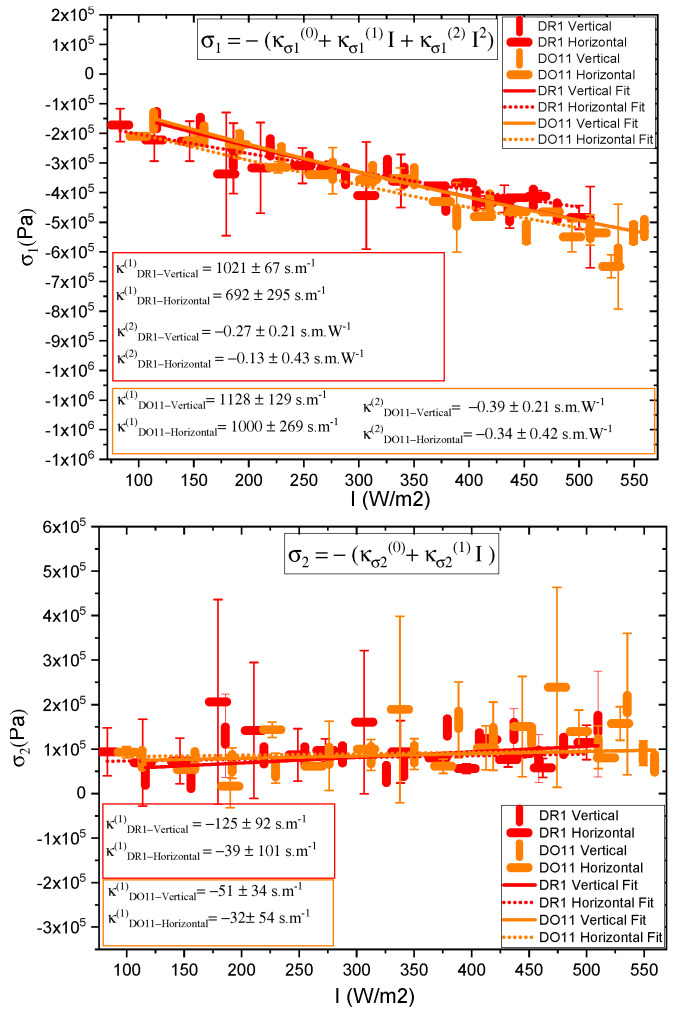
Amplitudes $\sigma_{1}$ (**top**) and $\sigma_{2}$ (**bottom**) for DR1- and DO11-Doped PMMA thin films as a function of pump intensity for both vertically and horizontally polarized pump. The negative stress of the fast process given by $\sigma_{1}$ implies that the length increases under light exposure and the increase gets larger with intensity. $\sigma_{2}$ is positive, implying that for the slow process the sample contracts when pumped with light.

The amplitudes of the photomechanical response for the fast and slow processes determined from Equation (2) are plotted in Figure 16. The faster response mechanism amplitude $\sigma_{1}$ shows a large dependence on the pump power, while the slower response associated with the amplitude $\sigma_{2}$ remains approximately unchanged as a function of the intensity.

The fits in Figure 16 to $\sigma_{1}$ and $\sigma_{2}$ as a function of intensity to the power series expansions

(37) $$\begin{array}{c} \sigma_{1}\left(I\right)=-\left(\kappa_{\sigma_{1}}^{\left(0\right)}+\kappa_{\sigma_{1}}^{\left(1\right)}I+\kappa_{\sigma_{1}}^{\left(2\right)}I^{2}\right) \end{array}$$

and

(38) $$\begin{array}{c} \sigma_{2}\left(I\right)=-\left(\kappa_{\sigma_{2}}^{\left(0\right)}+\kappa_{\sigma_{2}}^{\left(1\right)}I\right). \end{array}$$

Note that $\kappa_{\sigma_{1}}^{\left(0\right)}$ and $\kappa_{\sigma_{2}}^{\left(0\right)}$ are not the material constants but rather related to the pre-stress $\sigma_{0}$ when mounting the samples, which is given by

(39) $$\begin{array}{c} \sigma_{0}=\kappa_{\sigma_{1}}^{\left(0\right)}+\kappa_{\sigma_{2}}^{\left(0\right)}. \end{array}$$

Then, we can define an intensity-dependent photomechanical response as being given by

(40) $$\begin{array}{c} \kappa_{\sigma_{1}}^{\left(1\right)}\left(I\right)=\frac{\kappa_{\sigma_{1}}^{\left(0\right)}-\sigma_{1}\left(I\right)}{I}=\kappa_{\sigma_{1}}^{\left(1\right)}+\kappa_{\sigma_{1}}^{\left(2\right)}I. \end{array}$$

and

(41) $$\begin{array}{c} \kappa_{\sigma_{2}}^{\left(1\right)}\left(I\right)=\frac{\kappa_{\sigma_{2}}^{\left(0\right)}-\sigma_{2}\left(I\right)}{I}=\kappa_{\sigma_{2}}^{\left(1\right)}, \end{array}$$

where $\kappa_{\sigma_{i}}^{\left(1\right)}\left(I\right)$ is the long-time photomechanical stress response of process *i* to light of intensity *I*.

The parameters $\kappa_{\sigma_{1}}^{\left(1\right)}$ and $\kappa_{\sigma_{1}}^{\left(2\right)}$ are determined for DR1- and DO11-doped PMMA for both vertical and horizontal pump intensities from fits to the data to Equation (37) as shown in the top portion of Figure 16. The same is performed for the long-term stress response for the process associated with time constant $t_{2}$ by fitting the data in the bottom portion of Figure 16 to the power series expansion given by Equation (38). In this case, the data are relatively flat, so the fit includes only the linear term.

To summarize, the data given by Figure 16 are used to obtain the photomechanical coefficients $\kappa_{\sigma_{i}}^{\left(n\right)}$ for each process, which are substituted in Equations (37) and (38) to obtain the long-term stress $\sigma_{i}\left(I\right)$. These fits are also used to determine the intensity-dependent photomechanical response $\kappa_{\sigma_{i}}^{\left(1\right)}\left(I\right)$ for each process.

The strategy for isolating the mechanisms of the photomechanical response is to compare the photothermal heating model’s predictions given by Equation (9) with the magnitude of the fast and slow response determined from the data. The heating model depends on several types of parameters. First, $\sigma_{A}$, $\sigma_{B}$, $T_{0}$ and *n* characterize the thermodynamic properties of the material and are determined from the temperature-dependent stress of a clamped sample as shown in Figure 3. For DO11 and DR1, the data are fit to Equation (4) as shown in Figure 3 to obtain these parameters. Next, the physical properties of each sample, such as their thicknesses *w*, are directly determined with a micrometer. Properties such as the specific heat capacity of the polymer *c* and the material density ρ are sourced from the literature. We are thus assuming that the dopant dyes do not contribute substantially to these properties because of their low concentration. This assumption may need to be revisited given the large difference in the data between DO11 and DR1 in Figure 3. Finally, each of the time constants from the fits of the stress response are used to obtain τ at each intensity that is used in Equation (5) to relate the temperature change to the light intensity. Recall that $t_{1}\left(I\right)$ and $t_{2}\left(I\right)$ are defined to be the short and long time constants for pump intensity *I*. The strategy is to identify which exponential component of the two-exponential fit best agrees with the model’s prediction.

Alternatively, as we saw above, we can eliminate the parameter $\sigma_{B}$ in Equation (9) using Equation (4), yielding the semi-empirical model given by Equation (12). In this case, the measured stress $\sigma_{i}\left(I\right)$ at pump intensity *I* determined from the fits for the process of response time $t_{i}$ is used in the heating model, de-emphasizing reliance on the phenomenological stress model given by Equation (4). As we will see below, the small differences between these two approaches are insignificant in determining which process is associated with photothermal heating.

**Figure 17:**
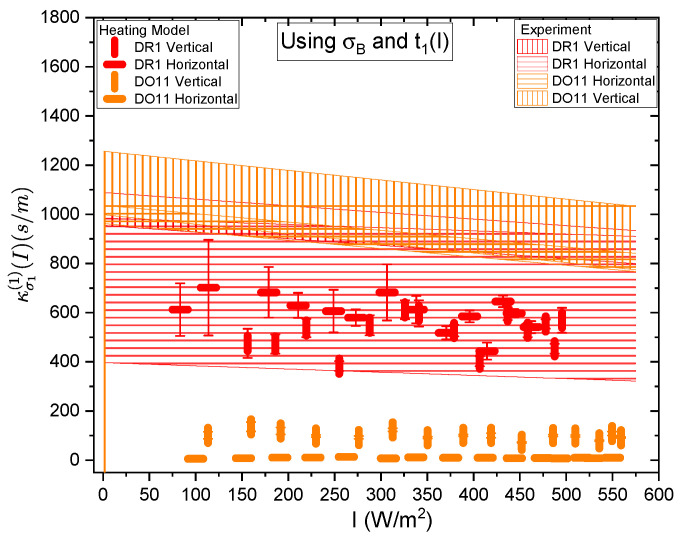
The vertical and horizontal lines (representing the pump polarization relative to the vertical) show the predicted heating contribution to the photomechanical response for DO11 and DR1 thin films using the theory given by Equation (9). The time constants $t_{1}\left(I\right)$ at each intensity used in the model are the measured ones in Figure A1 in Appendix A. The width of the bands show one standard deviation band of the magnitude of the fast component of the experimentally determined intensity dependent photomechanical constant, which is determined from a quadratic fit of the stress data shown in the top graph in Figure 16.

**Figure 18:**
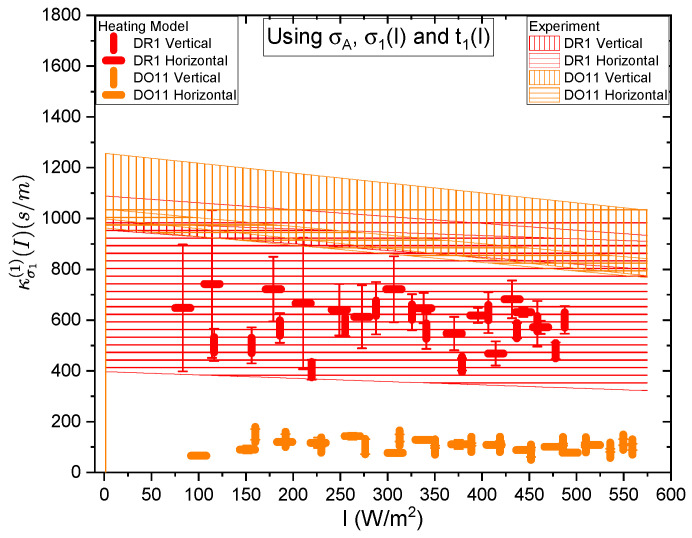
The vertical and horizontal lines (representing the pump polarization relative to the vertical) show the predicted heating contribution to the photomechanical response for DO11 and DR1 thin films using $t_{1}$ for the time constant as a function of intensity using the semi-empirical theory given by Equation (12). The one-standard-deviation bands show the experimentally determined intensity-dependent photomechanical constant using a fit of the stress data to a quadratic function in the intensity, as shown in the top graph in Figure 16.

**Figure 19:**
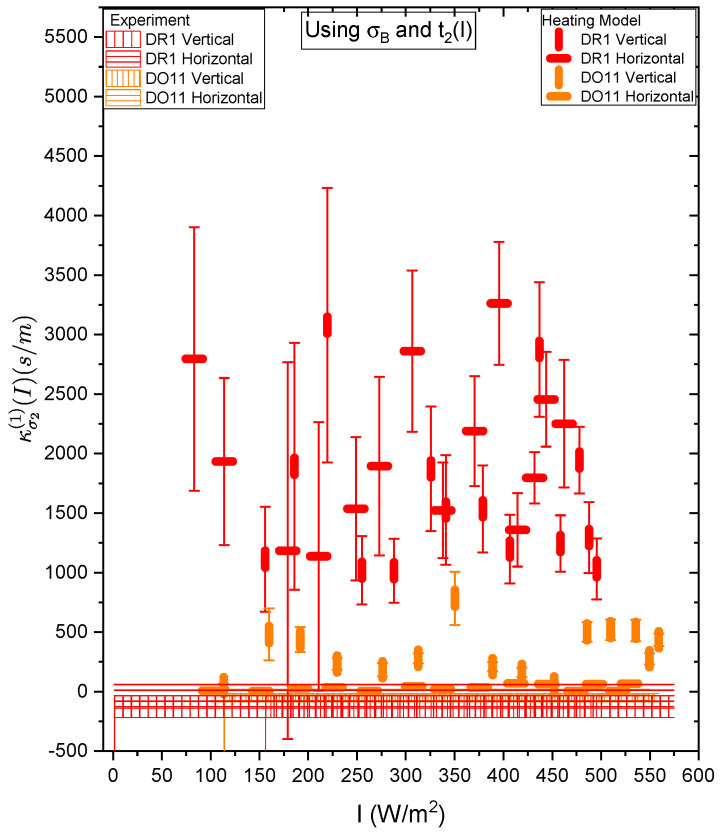
The vertical and horizontal lines (representing the pump polarization relative to the vertical) show the predicted heating contribution to the photomechanical response for DO11 and DR1 thin film using $t_{2}\left(I\right)$ as the time constant in the model given by Equation (9). The bands show the experimentally determined intensity-dependent photomechanical constant determined by the fit of the stress data to a quadratic function in the intensity, as shown in the bottom graph of Figure 16.

**Figure 20:**
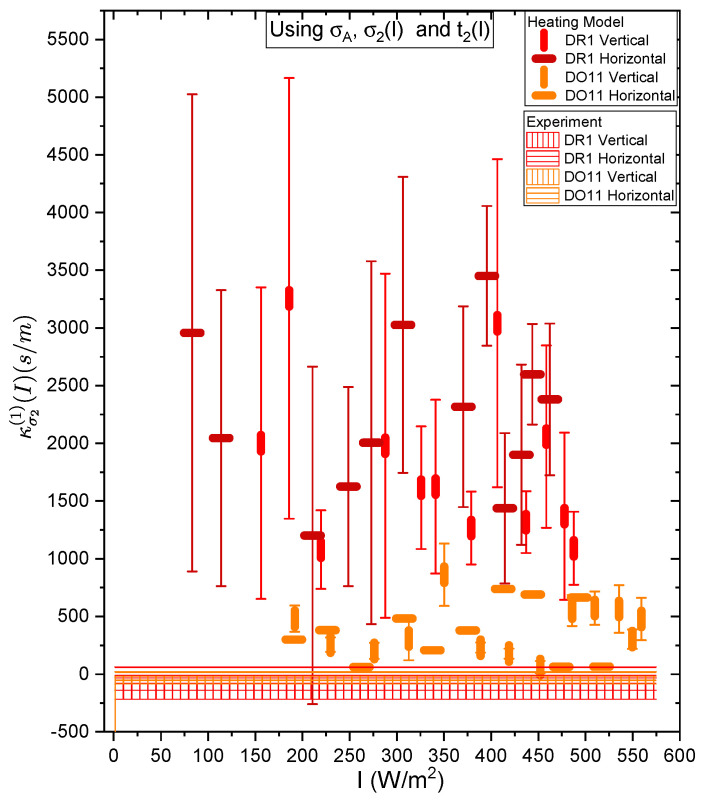
The vertical and horizontal lines (representing the pump polarization relative to the vertical) show the predicted heating contribution to the photomechanical response for DO11 and DR1 thin film using $t_{2}\left(I\right)$ as the time constant in the semi-empirical model given by Equation (12). The one-standard-deviation error bands show the experimentally determined intensity-dependent photomechanical constant determined from the fit of the stress data to a quadratic function in the intensity, as shown in the bottom graph of Figure 16.

Figure 17 and Figure 18 show a summary of the theoretical and experimental intensity-dependent photomechanical constant $\kappa_{\sigma_{1}}\left(I\right)$ for the faster process with response time $t_{1}$. The bands are derived from the experimentally determined amplitudes of the fast process and their widths represent the one standard deviation uncertainty band. The vertical and horizonal data markers represent the photothermal heating model’s prediction for the intensity-dependent photomechanical response for the two orthogonal pump polarization directions. Their values are determined using the heating theory given by Equation (9), plotted in Figure 17, and Equation (12), plotted in Figure 18. The scatter in the plots originate from the scatter in the measured time constants $t_{1}\left(I\right)$ that are input parameters to the theory. The semi-empirical theory using Equation (12) has an additional contribution to the uncertainty from $\sigma_{1}\left(I\right)$ and $\sigma_{A}$, which leads to larger error bars. Similarly, the error bands of the data are coded with vertical and horizontal hash marks to represent the polarization direction. Figure 19 and Figure 20 show the slower process with the time constant data and theory using $t_{2}\left(I\right)$ and $\sigma_{2}\left(I\right)$ for the analysis.

Figure 17 and Figure 18 show that the magnitude of the measured fast linear photomechanical response, as represented by the bands, decreases with pump intensity. The samples are pristine at the start of the experiment and characterized in the same physical spot starting with low intensity and then ending at the highest intensity with uniform intensity increases in between. Measurements of the order parameter, as discussed above, show that the material undergoes long-term changes in axial order of the dopant dyes, which we provisionally attribute to long-term molecular reorientation. This change in the fast response with exposure is consistent with the long-term process of molecular reorientation. Figure 17 and Figure 18 show that the data and the photothermal heating theory for the fast response agree for DR1 but disagree for DO11. As such, we conclude that the fast process is due to heating in DR1 but not in DO11. This is puzzling unless our assumption that the heat capacities are the same for both materials is wrong. The data suggest that the heat capacity of DO11 would need to be an order of magnitude smaller than in neat PMMA to make the heating theory agree with the data, which seems unlikely.

Figure 19 and Figure 20 show the experimental data bands and the theory markers for the slower response given by time constant $t_{2}$. For DO11, the photomechanical response of the data and theory seem to marginally agree with some outliers, while for DR1, the data disagree with theory. However, the signs disagree. This suggests that the slow response is not associated with photothermal heating in both materials.

Table 4 summarizes the data. The fast response time of both DR1- and DO11-doped PMMA thin films is the same within experimental uncertainty and is consistent with the time constant being determined from the sample geometry, which determines how heat dissipates. For heating, then, the time constants are expected to be similar, as is $t_{1}$. The fact that the time constants are independent of polarization is also what one would expect from an isotopic sample. This gives us greater confidence that the fast response originates in photothermal heating. The fact that DO11 has a smaller linear response from heating is also consistent with Figure 3, which shows that heating results in a smaller temperature-dependent force. All of these data together are consistent with photothermal heating being responsible for the fast process.

The slow response in both materials is of negative sign, consistent with sample contraction as one would expect for molecular hole burning and molecular reorientation away from the pump polarization direction. Molecular reorientation would result in a positive response for a perpendicular pump beam, while orientational hole burning would give a negative response, but of smaller magnitude. The data are consistent with hole burning, but the uncertainties are too large to make a definitive conclusion.

The critical intensity $I_{{\mathrm{cr}}_{1}}$ due to the fast heating process is defined by

(42) $$\begin{array}{c} I_{{\mathrm{cr}}_{1}}=\left|\frac{\kappa_{\sigma_{1}}^{\left(1\right)}}{\kappa_{\sigma_{1}}^{\left(2\right)}}\right|, \end{array}$$

and quantifies the intensity at which the higher-order term exceeds the linear response. DO11’s critical intensity is smaller than DR1’s value because its linear response is smaller. For both materials and both polarizations, the first nonlinear correction to the photomechanical response $\kappa_{\sigma_{1}}^{\left(2\right)}$ is the same within experimental uncertainty.

## 6. Conclusions

This paper presents the results of extensive studies of the time- and polarization-dependent photomechanical response of thin films made of PMMA polymer doped with (1) DR1 and (2) DO11 dyes. In addition, dichroism measurements are used to determine the degree of hole burning and the evolution of molecular orientation due to both the short-time and reversible response to the pump beam and long-term quasi-irreversible reorientation due to prolonged-exposure. Also measured is the temperature-dependent stress of a clamped sample to determine its thermodynamic equation of state, which is used to build a model of the photothermal photomechanical response.

Time-dependent dichroism measurements probe the dopant dye’s orientational distribution function in response to a strong pump being turned on and off. In DR1-doped PMMA, these measurements show a fast evolution of dichroism due to hole burning and a slower but stronger signal due to molecular reorientation. These observations are consistent with expectations of a fast initial excitation of a cis isomer followed by the slower process of reorientational diffusion. DO11, which does not photoisomerize, shows no long-term molecular reorientation but does show a smaller degree of hole burning. The underlying mechanism is most likely due to the photo-tautomerization due to a proton hopping from the amine group to the carbonyl group, which is a known process responsible for self-healing [36,37,38].

Intensity-dependent dichroism measurements are used to determine the axial order parameter $\langle P\rangle$, which is a measure of the axial alignment of the dopant molecules. In DR1, the pristine material after fabrication is slightly anisotropic. The observed dichroism after the material reaches equilibrium under optical pumping increases with increased intensity, as we would expect of molecular reorientation where the reorientational diffusion rate increases with pump intensity. However, the 15 s recovery time between measurements is not enough time to allow for the reorientational alignment to relax. At the higher intensities, the residual axial alignment with the pump off in the time interval that precedes the pump can be as high as 40% of the value induced when the pump is turned on. This residual alignment builds over the course of the experiment. As such, the fraction of the pump light absorbed decreases since fewer molecules have a substantial projection of their axis along the pump polarization. This will lead to a smaller photomechanical response than for a pristine sample. In contrast, DO11 is isotropic from the start and shows very few intensity-dependent effects.

The photomechanical experiments measure the stress induced by light for a clamped sample for two pump polarizations. This can be viewed as the photobaric analogy of photochromism, where light-polarization-dependent stress changes take the place of light-polarization-dependent color change. These measurements show that photothermal heating is the dominant mechanism, with the others falling below experimental uncertainties. The conclusion is that the dye molecules couple weakly to the polymer so that they do not affect its photomechanical properties. In dye-doped polymers, then, the role of the dopant molecules is to absorb light and transfer the energy as heat to the polymer, which reacts through a thermodynamic process of thermal expansion.

In a parallel tract, we have developed a heating model that predicts the pure photothermal heating response. The critical input parameters to this model are the materials’ thermodynamic and mechanical properties. The thermally induced stress in the clamped configuration is a parameter that is difficult to determine from first principles. As such, we have measured it directly and fit it to an empirically determined analytical model with three adjustable parameters, which fully characterizes the thermomechanical properties of the material. Surprisingly, the parameters for DO11- and DR1-doped PMMA are vastly different, showing that the small concentration of dopants can vastly change these properties.

Finally, we have used the thermomechanical parameters to predict the photomechanical response. This allows for a more fine-tuned experiment to separate the mechanisms. A fit to the photomechanical data of DR1 to a double exponential separates the fast from the slow response, and a comparison to the heating theory shows that the fast response is due to heating and the slow response is an order of magnitude smaller than the heating response, but within uncertainty of being zero. It is a puzzle why DO11 does not fit the heating theory even though the magnitude of the measured response is the same as in DR1. This may be due to the fact that the photobaric data of DR1 and DO11 are so different. This remains the topic of future research.

## Figures and Tables

**Table 1:** The parameters determined by fitting the temperature-dependent stress data to Equation (4) for DR1- and DO11-doped PMMA thin films.

Sample	$\sigma_{A}$ (MPa)	$\sigma_{B}$ (MPa)	$T_{0}$ (°K)	n
DR1/PMMA thin film	−300 (±0)	300 (±0.002)	367.88 (±0.28)	32.40 (±0.14)
DO11/PMMA thin film	−300 (±0)	300 (±0.0004)	344.95 (±0.18)	57.97 (±0.24)

**Table 2:** A summary of each mechanism’s contribution to the photomechanical response in dye-doped PMMA thin films. Fiber data are from the literature, refs. [2,33], where “CTA” refers to the concentration of charge transfer agent, which affects the polymer’s chain length.

Sample	$\kappa_{//}^{\left(1\right)}/\kappa_{⊥}^{\left(1\right)}$	*R*	$\kappa_{//}^{\left(\mathrm{heat}\right)}=\kappa_{⊥}^{\left(\mathrm{heat}\right)}$ (s/m)	$\kappa_{//}^{\left(\mathrm{hole}\right)}$ (s/m)	$\kappa_{⊥}^{\left(\mathrm{hole}\right)}$ (s/m)
DR1 Thin Film	0.92 ± 0.12	−0.06 ± 0.080	961.35 ± 124.91	−54.61 ± 77.86	27.31 ± 38.88
DO11 Thin Film	0.97 ± 0.17	−0.02 ± 0.12	841.07± 106.23	−18.21 ± 98.43	9.11 ± 49.20
DR1 Fiber 1.33 × 10^−3^ CTA	0.96 ± 0.28	−0.02 ± 0.19	246 ± 80	−6 ± 46	3 ± 23
DR1 Fiber 2 × 10^−3^ CTA	0.91 ± 0.08	−0.06 ± 0.05	259 ± 22	−15 ± 14	8 ± 7
DR1 Fiber 2.66 × 10^−3^ CTA	0.94 ± 0.23	−0.04 ± 0.16	231 ± 49	−10 ± 36	5 ± 18

**Table 3:** A summary of the results for both thin films. Fiber data are from the literature [2].

Material	Polarization	$\kappa_{\sigma }^{\left(1\right)}$ (s/m)	$\kappa_{\sigma }^{\left(2\right)}$ (s·m/W^2^)	E (Gpa)	FOM ($10^{-4}$ s^2^/N)
DR1 Thin Film	Vertical	995 ± 161	−0.21 ± 0.27	0.89 ± 0.01	11.12 ± 1.80
DR1 Thin Film	Horizontal	828 ± 204	0.12 ± 0.31	0.89 ± 0.01	7.7 ± 1.809
DO11 Thin Film	Vertical	1135 ± 201	−0.28 ± 0.28	0.89 ± 0.01	14.50 ± 2.57
DO11 Thin Film	Horizontal	1150 ± 178	−0.25 ± 0.27	0.89 ± 0.01	14.90 ± 1.02
DR1 Fiber 1.33 × 10^−3^ CTA	Vertical	240 ± 63	−5 ± 8	3.12 ± 0.05	0.19 ± 0.05
DR1 Fiber 1.33 × 10^−3^ CTA	Horizontal	249 ± 35	−2 ± 0.03	3.12 ± 0.05	0.20 ± 0.03
DR1 Fiber 2 × 10^−3^ CTA	Vertical	243 ± 15	−5 ± 3	3.02 ± 0.04	0.20 ± 0.01
DR1 Fiber 2 × 10^−3^ CTA	Horizontal	266 ± 16	−10 ± 2	3.02 ± 0.04	0.23 ± 0.01
DR1 Fiber 2.66 × 10^−3^ CTA	Vertical	221 ± 30	−2 ± 4	2.87 ± 0.02	0.17 ± 0.02
DR1 Fiber 2.66 × 10^−3^ CTA	Horizontal	235 ± 48	−13 ± 4	2.87 ± 0.02	0.19 ± 0.04
DR1 Fiber 2.66 × 10^−3^ CTA	Vertical	263 ± 42	6 ± 4	2.87 ± 0.02	0.24 ± 0.08
DR1 Fiber 2.66 × 10^−3^ CTA	Horizontal	266 ± 20	1.3 ± 3	2.87 ± 0.02	0.25 ± 0.04

**Table 4:** A summary of the photomechanical constants for each process, their time constants and the critical intensity for vertical (V) and horizontal (H) pump polarization.

Material	$\kappa_{\sigma_{1}}^{\left(1\right)}$ (s/m)	$\kappa_{\sigma_{1}}^{\left(2\right)}$ (s·m/W)	$\kappa_{\sigma_{2}}^{\left(1\right)}$ (s/m)	$t_{1,\mathrm{avg}}$ (s)	$t_{2,\mathrm{avg}}$ (s)	$I_{{\mathrm{cr}}_{1}}\left(W\cdot m^{-2}\right)$
DR1 (V)	1021 ± 67	−0.27 ± 0.21	−125 ± 92	0.500 ± 0.018	1.56 ± 0.17	3785.19 ± 2954
DR1 (H)	692 ± 295	−0.13 ± 0.43	−39 ± 101	0.582 ± 0.018	1.94 ± 0.17	5323.1 ± 17,752
DO11 (V)	1128 ± 129	−0.39 ± 0.21	−51 ± 34	0.500 ± 0.021	1.68± 0.26	2892.31 ± 1592
DO11 (H)	1000 ± 269	−0.34 ± 0.42	−32 ± 54	0.462 ± 0.024	1.56 ± 0.32	2941.2 ± 3718

## Data Availability

The original contributions presented in this study are included in the article. Further inquiries can be directed to the corresponding author.

## Abbreviations

The following abbreviations are used in this manuscript:

DR1	Disperse Red 1
DO11	Disperse Orange 11
PMMA	Poly Methyl Methacrylate
FOM	Figure of Merit
PGMEA	Propylene Glycol Methyl Ether Acetate
PBS	Polarizing Beam Splitter

## Appendix A

This appendix includes data, in the form of plots, that are used in the main text for the heating model.
